# Green Synthesis of Substituted Anilines and Quinazolines from Isatoic Anhydride-8-amide

**DOI:** 10.1038/s41598-019-50776-y

**Published:** 2019-10-03

**Authors:** Sudershan R. Gondi, Asim K. Bera, Kenneth D. Westover

**Affiliations:** Departments of Biochemistry and Radiation Oncology, The University of Texas Southwestern Medical Center at Dallas, Dallas, Texas 75390 United States

**Keywords:** Drug discovery, Cancer therapy

## Abstract

Synthetic methods used to generate substituted anilines and quinazolines, both privileged pharmacological structures, are cumbersome, hazardous or, in some cases, unavailable. We developed a straightforward method for making isatoic anhydride-8-amide from isatin-7-carboxylic acid as a tool to easily produce a range of quinazoline and substituted aniline derivatives using adaptable pH-sensitive cyclization chemistry. The approaches are inexpensive, simple, fast, efficient at room temperature and scalable, enabling the synthesis of both established and new quinazolines and also highly substituted anilines including cyano derivatives.

## Introduction

The 1,3-diazanaphthalene, or quinazoline, structural motif is a privileged pharmacological scaffold^1^ found in a wide range of bioactive compounds designed for treating health conditions including cancer, inflammation, hypertension, obesity, and infection^2–6^. Similarly substituted anilines are key elements of bioactive compounds^7^ and valuable industrial reagents^8,9^ (Fig. S1). Current synthetic methods involve using costly or hazardous reagents under harsh conditions to functionalize quinazolines and substituted anilines at late stages in synthetic schemes, and also require labor-intensive purification of chemical intermediates^10,11^. Moreover, options for functionalizing these structures are limited and challenging^12^.

## Easy Synthesis of IAA

**Figure 1:**
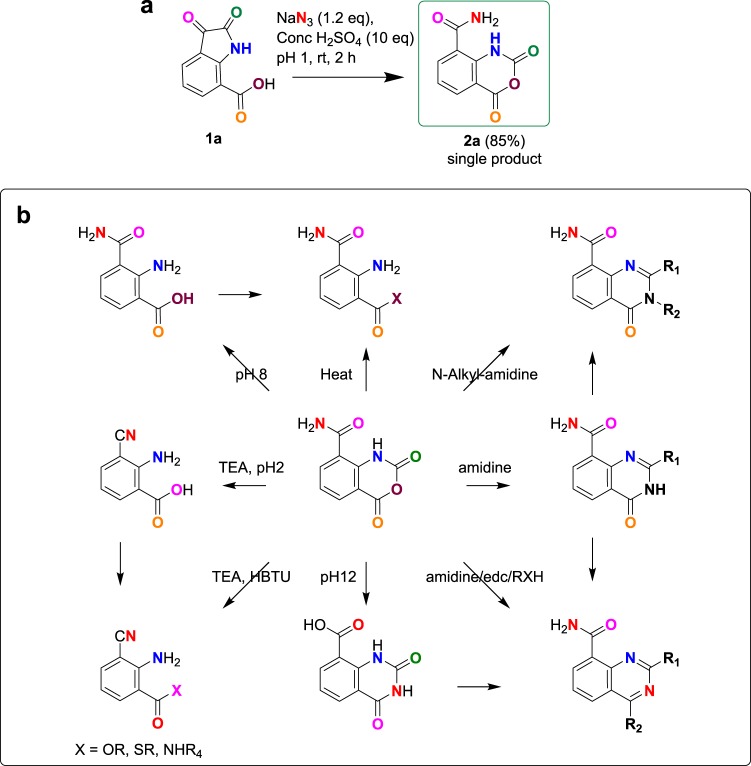
(**a**) Synthesis of IAA. (**b**) Roadmap for synthesis of substituted anilines and quinazolines from IAA.

We developed a straightforward, broadly applicable, environmentally conscious method to prepare quinazoline and anilines analogues via isatoic anhydride-8-amide (IAA) after observing that IAA can be prepared directly using the Schmidt reaction from 2,3-dioxoindoline-7-carboxylic acid **1a**, also known as isatin-7-carboxylic acid, which can be obtained from anthranilic acid^13^. The reaction occurred efficiently at room temperatures in sulfuric acid and sodium azide in yields of ~85% (Fig. 1a). Under these conditions, IAA **2a** precipitates, eliminating the need for additional purification or workup. This result surprised us because previous reports showed that treatment of isatin-7-carboxylic acid with sulfuric acid produces multiple products and, therefore, is unsuitable for synthetic workflows^14^. Nevertheless, we confirmed the identity of IAA as a single product by mass spectrometry, NMR, and x-ray crystallography (Fig. S2 and Table S1). We found that the reaction also occurred in yields of 50–75% with other azide sources such as tetrabutylammonium azide and tosyl azide, wherein *in situ* production of hydrazoic acid improves the safety of the reaction. However, sources such as acyl azide, alkyl azide, and alkoxy acyl azide were less efficient or did not produce IAA (Table 1). To our knowledge this is the first example of using tosyl azide to yield benzoxazine. Alternative acids such as hydrochloric acid yielded no product, suggesting that sulfuric acid is critical to the mechanism. Reactions in DMSO produced isatin 7 acid-2-azide **2a2**, suggesting that azide ions attack the C2-amide rather than the C3 carbonyl group without sulfuric acid (Table 1, **entry 7**). We found that IAA is an important intermediate for synthesizing a number of substituted anilines and quinazolines (Fig. 1b).

**Table 1:** IAA reaction optimization.


**Entry**	**Azide source**	**Solvent**	**Yield (%)**
1	Sodium Azide	H_2_SO_4_	85
2	Tosyl Azide	H_2_SO_4_	75
3	Tetrabutyl Azide	H_2_SO_4_	50
4	RCON_3_	H_2_SO_4_	5
5	ROCON_3_	H_2_SO_4_	0
6	RN_3_	H_2_SO_4_	0
7	Sodium Azide	DMSO	0
8	Sodium Azide	HCl	0

## Mechanism of IAA Formation

We thought that IAA may form through a mechanism other than a typical Schmidt reaction for a ketone or free acid, wherein alkyl migration is accompanied by loss of carboxylic acid yielding an amine^15^. Instead, the mechanism occurred by solvolysis between the 2, 3 position, allowing direct azidation of the C3 keto of isatin-7-carboxylic acid and leading to a rightward (clockwise) cyclization between the NH-carbonyl and 7-acid to give isatoic anhydride (Fig. S3a). To confirm this mechanism, we evaluated isatin analogues with substitutions at position 4, which we expected to compete for interactions with the amide intermediate in a counterclockwise (leftward) cyclization reaction, leading to the formation of phthalimide compounds (Fig. S3b). Consistent with this model, isatin-4-acid **1b**, methyl ester **1c**, primary amide **1d**, C3-hydrazide **1e**, and alkyl substitution at position 1 **1f**, **1g** produced 3-amino-phthalimide **2b** or 3-methylamino-phthalimide **2f** (Table 2a, **entries 1–6**). As expected, moving the acid to the fifth position **1h** prevents cyclization (Table 2a, **entry 7**). In addition to supporting the proposed IAA mechanism, we also showed for the first time how phthalimides, used in the synthesis of quinazoline 5-acid derivatives from an isatin derivative, are formed^16^. These sulfuric acid-based methods are easier to perform than established procedures for generating phthalimides involving urea^17^, ammonium carbonate at elevated temperature, or reduction using palladium^18^. This method also provides synthetic pathways to a wide range of N-substituted phthalimides, whereas prior methods are limited to 3-methylamino-phthalimide^17,19^.

**Table 2:**
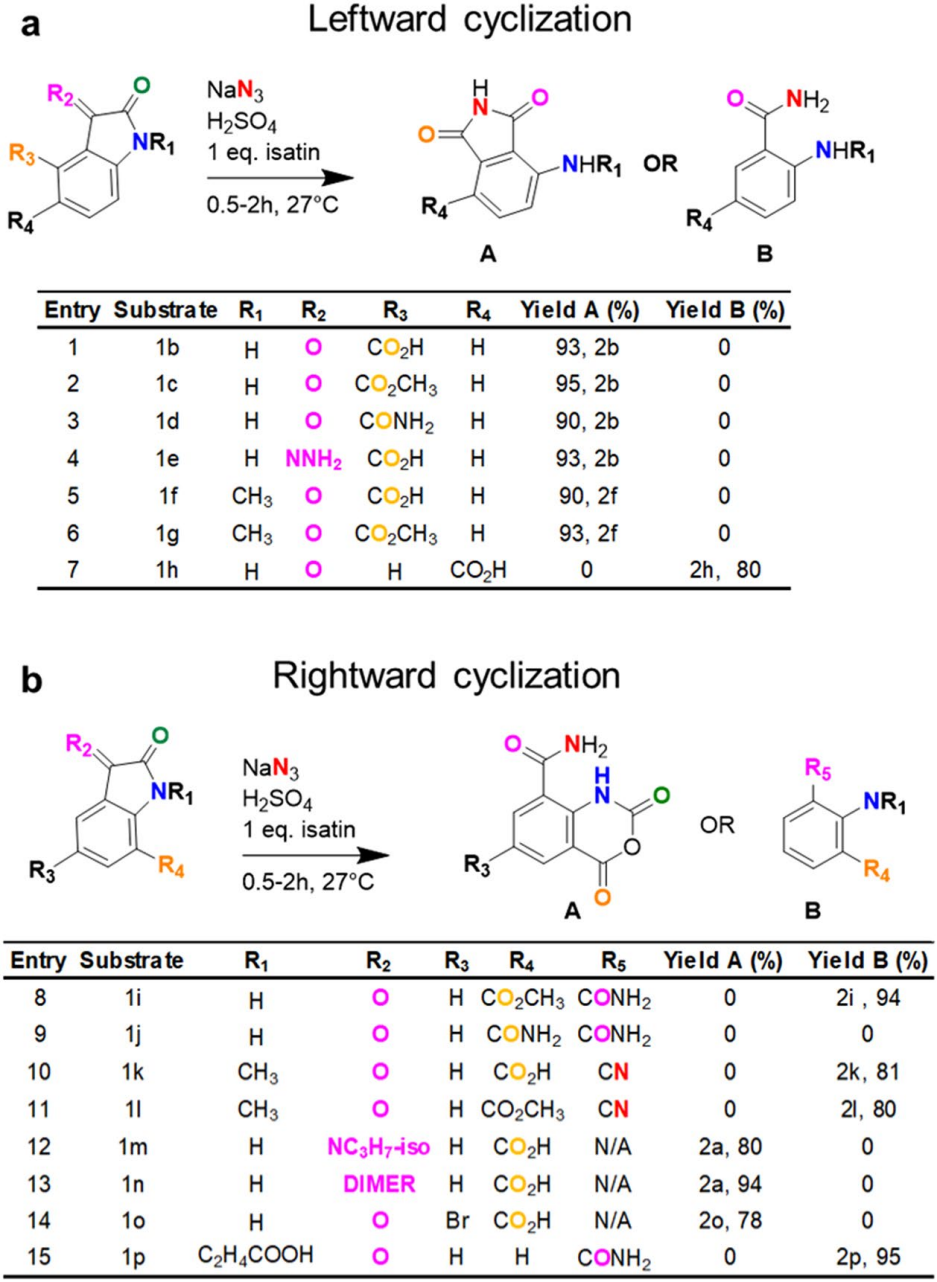
IAA formation mechanism.

While it is theoretically possible that the clockwise (rightward) cyclization required to form IAA may occur with either an acid, ester, or amide, the acid at position 7 is preferred (Table 2b, **entries 8–9**). Cyclization also depends on the availability of the secondary amine at position 1, as shown by failure to produce  N-alkyl IAA when an alkyl group is introduced at position 1 (Table 2b, **entry 10**, **11**). Surprisingly, alkyl substitution at first position yielded a cyano aniline for both acid and esters **2k**, **2l** (Table 2b, **entries 10**, **11**), suggesting that the combined effects of electron withdrawal by the acid and N-alkyl groups contribute to forming nitrile from the primary amide. Of note, known methods for the synthesis of n-alkylated nitrile-acid nitrile-ester involve using corrosive chemicals, high temperatures, and purification composed of multistep reactions, which are eliminated with the current method^20^. To assess whether incorporating a secondary amide at position 8 is possible, we started with enamines at position 3 **1m**, **1n**, although they yielded IAA instead of a secondary amide (Table 2b, **entries 12**, **13**). This finding suggested that enamines are unstable under acidic conditions. Substitution on the phenyl ring with bromine at position 5 **1o** was also tolerated, giving **2o** in excellent yield (Table 2b, **entry 14**). To exclude the possibility that cyclization may occur using an n-alkyl acid at position 1 we tested reactions with isatin-N-propanoic acid and obtained an open chain product **2p** (Table 2b, **entry 15**), instead of oxazine, suggesting that oxazine is less stable than benzoxazine.

Our synthetic method simplifies isatin transformations considerably compared to prior methods. Synthesis of isatoic anhydride from isatin previously required using reagents such as peroxides^21,22^, phenyliodide^23^, and NBS^24^; making benzamide derivatives from isatin required using chromic acid^25^, peroxide/phosphate buffer systems^26^, or *in situ* generated hydrazoic acid^27,14^; and generating amino benzamide derivatives from isatoic anhydride required using ammonia^22,28^ and ammonium carbonate^29^. In contrast, we can now approach multiple classes of derivatives from IAA using mild, established reactions.

## Scope of Substituted Anilines Originating from IAA

We recognized that IAA could be used as a starting material for producing a range of useful substituted anilines and quinazoline derivatives *in situ*, primarily by manipulating pH, with either neutral or acidic conditions giving substituted anilines where position 1 (Table 3, **R**_**1**_) can include a range of substituents including acids, esters, thioesters, amides, and halogens. Conversion of IAA to the open chain 3-acid-2-amino-benzamide **3a** is readily accomplished at pH 7 and also by heating in sulfuric acid (Table 3, **entry 1**). Next, the corresponding ester, 3-ethylester, 2-amino-1-bezamide **4a** was prepared by allowing IAA to react with polar solvents (ethanol) under reflux conditions (Table 3, **entry 2**). Alternatively, esters can be achieved by allowing IAA to react with K_2_CO_3_ in a polar solvent at room temperature. We showed that multiple ester derivatives **4a**, **4b** including a thioester **5** could be obtained using this method (Table 3, **entry 3**).

**Table 3:**
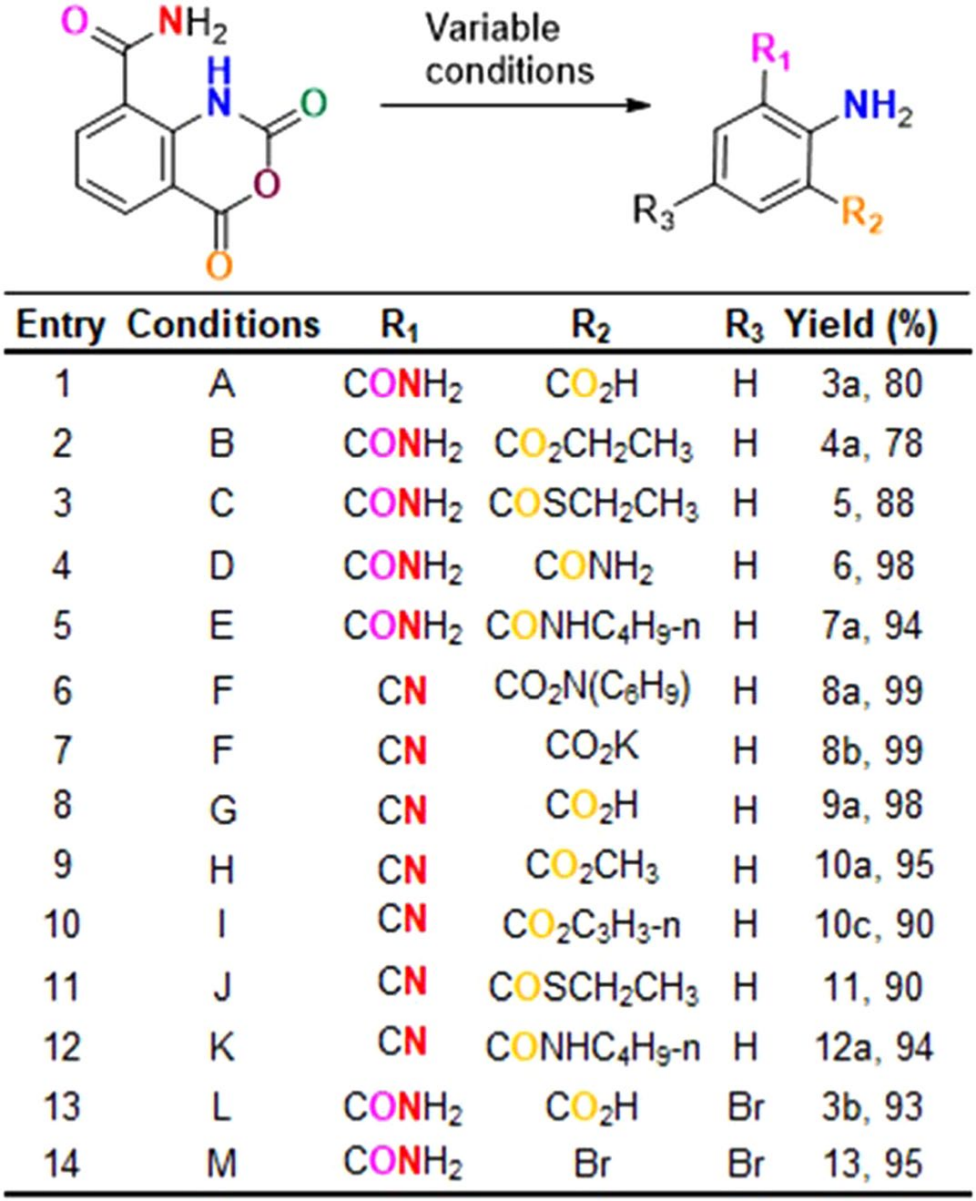
Scope of aniline chemistry. Conditions are as follows: (A) NaOH, 0 °C, 10 min. (B) MeOH, reflux, 2 h. (C) K_2_CO_3_, DMF, EtSH, 27 °C, 6 h. (D) (NH_4_)_2_CO_3_, Dioxane, 60 °C, 4 h. (E) n-C_4_H_9_NH_2_, DMF, 50 °C, 6 h. (F) K_2_CO_3_/TEA, DMF, 50 °C, 3 h. (G) K_2_CO_3_/TEA, DMF, 50 °C, 3 h. (H) K_2_CO_3_, DMF, 50 °C, 3 h, MeI, rt, 12 h. (I) TEA, HBTU, Propargyl-OH. 12 h. (J) TEA, HBTU, EtSH, 12 h. (K) TEA, HBTU, n-C_4_H_9_NH_2_, 12 h. (L) 1.0 eq Bromine, H_2_SO_4_, 100 °C, 10 min. (M) 2.0 eq Bromine, H_2_SO_4_, 100 °C, 10 min.

Because transformation of isatoic anhydride to 2-amino-benzamide using ammonium carbonate in dioxane was previously reported^29^, IAA was treated under these conditions giving 2-amino, 1,3 benzadiamde, in quantitative yield **6** (Table 3, **entry 4**). The same product can also be obtained by treating IAA with ammonium carbonate or ammonium acetate in DMF or DMSO. These methods are simpler than established procedures involving CrO_3_, SOCl_2_, ammonia, and palladium or Raney Nickel^7^. We next attempted to generate non-symmetrical amides by treating with primary amines and achieved a series of derivatives in 80–90% yield (Table 3, **entry 5**, Supplement 7a–g).

Surprisingly, when we treated IAA with secondary or tertiary amines, we obtained a cyano- salt **8a** (Table 3, **entry 6**). In another reaction we used K_2_CO_3_ to neutralize the amine salts and found that these also provided a cyano-salt **8b** (Table 3, **entries 7**). These two reactions suggested that a range of cyano derivatives could be obtained from IAA. The reaction likely proceeds by conversion of the primary amide to a nitrile, leading to the *in situ* generation of KOH, which hydrolyzes isatoic anhydride to give a 1-cyano-2-amine 3-acid-potassium salt. This, in turn, gives a cyano HSA **9a**, quenching within the acidic medium (Table 3, **entries 8**, Fig. S4). We could elaborate on this series at the third position. Treating with alkyl halide overnight at room temperature gave the corresponding cyano-ester **10** (Table 3, **entry 9** and Supplement 10a–e). In the same way, *in situ*-generated cyano-TEA salt in the setting of alkylhalide treatment gave cyano-ester (Table 3, **entry 10** and Supplement 10c,e), cyano-thiol ester **11** amide (Table 3, **entry 11**), and cyano-amide (Table 3, **entry 12** and Supplement 12a–g), by treating with corresponding alcohol, thiol, and amines in the presence of coupling reagents.

Finally, we speculated that additional functionalization could also be obtained through halogenation, allowing us to introduce new substituents through halide-dependent coupling reactions. We accomplished this by combining IAA with 1 equivalent of liquid bromine in concentrated sulfuric acid. This yielded 5-bromo **3b** (Table 3, **entry 13**). However, with 2 equivalents of liquid bromine we obtained 2-amino-4,6 dibromo-benzamide **13** (Table 3, **entry 14**)^30^. This is the first report of decarboxylation of isatoic anhydride with a halogen.

## Scope of Quinazolines Originating from IAA

We next focused on the conversion of IAA to quinazoline derivatives, (Table 4). IAA readily transformed to quinazoline-8-carboxylic acid **14a** by heating or exposure to basic conditions (Table 4, **entry 1**). Interestingly, while attempting to make tertiary butyl esters from IAA by using potassium tertiary butoxide, we instead obtained either **14a** at room temperature or **9a** at 50 °C, indicating the tunability and versatility of the IAA reactions. The corresponding quinazoline-8-amide **15** was achieved by adding amine and coupling reagent to *in situ* generated quinazoline-8-acid at 100 °C (Table 4, **entry 2**). To show that N3-substituted quinazoline can be obtained, we treated IAA with an amine followed by PhNCS, yielding an N3-substituted quinazoline **16** (Table 4, **entry 3**). Alternatively, N3-substituted quinazoline **17** was obtained by treating with 1 equivalent of an amine source in acetic acid, suggesting that the secondary amide is preferred over primary amide during cyclization (Table 4, **entry 4**). An attempt to convert p-amide to nitrile using TFAA in pyridine unexpectedly gave a 2-substituted quinazoline **18**, showing that amide is preferred over acid during cyclization and also indicating the possibility of generating a series of 2-substituted quinazolines. (Table 4, **entry 5**).

**Table 4:**
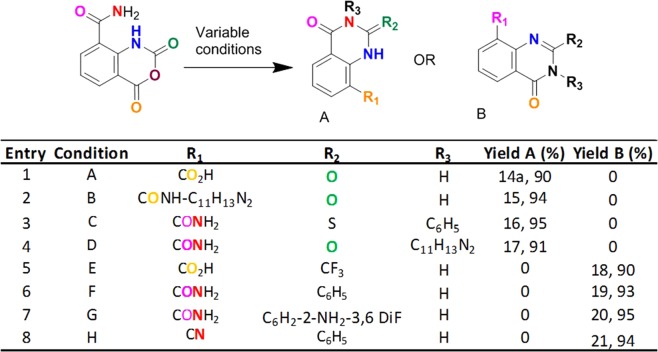
Scope of Quinazoline chemistry. (A) NaOH, 0 °C, 10 min. (B) 100 °C, 1 h, HBTU, 2-Aminobenzimidazole, 12 h. (C) n-C_4_H_9_NH_2_, 50 °C 3 h, PhNCS, 50 °C, 6 h. (D) 2-Aminobenzimidazole, AcOH, 100 °C 12 h. (E) TFAA, pyridine, 27 °C, 6 h. (F) Benzamidine/N-Phenylbenzamidine, 42 °C, 6 h. (G) 2-Amino-3,5-diflouoro-benzamidine-HCl, K_3_PO_4_, 80 °C, 2 h. (H)TEA, 50 °C, 3 h, HBTU, Benzamidine, 12 h.

We noted that a number of quinazoline-based inhibitors, including inhibitors of BRAF and p38, utilize C2 and N3 substitutions^31^. We speculated that these substitutions could be obtained by reactions with amidine-containing compounds, if they react at C4. Accordingly, the combination of benzimidine with IAA resulted in 2-substituted quinazolines-8-amide **19** in excellent yields (Table 4, **entry 6**). *N*-substituted benzamidine also leads to isolating **19**, indicating selectivity of primary amide formation for secondary amide during cyclization. Surprisingly, 2-amino benzamidine gave a similar product **20**, showing selectivity of benzimidine-NH_2_ over phenyl-NH_2_ (Table 4, **entry 7**). The corresponding quinazoline-8-cyano derivatives **21** can be synthesized by treating *in situ*-generated nitrile-TEA salt with amines and coupling reagents (Table 4, **entry 8**).

Developing IAA and its derivatives opens new options for generating highly substituted anilines and functionalized quinazolines. The versatility is multiplied by the availability of many anthranilic acid derivatives, allowing to easily generate IAA derivatives that contain functional group positions 4, 5, and 6, which may lead to generating quinazolines functionalized in the 5, 6, and 7 positions (Fig. S5); a challenging and perhaps impossible transformation at the quinazoline stage. Likewise, a few examples of anilines with 3 ortho groups, either as intermediates or end products, are found in the literature. This raises the prospect of generating novel highly substituted anilines with functional groups that were not previously considered in medicinal and industrial chemistry.

## Materials and Methods

### Chemicals

Starting materials, reagents, and solvents were purchased from commercial sources and used as received, unless stated otherwise. Isatin derivatives were purchased from Enamine, a Sigma-Aldrich partner. Melting points were determined using μTherm°Cal_10_ (Analab scientific Pvt. Ltd.) melting point apparatus and are not corrected. Reaction progress was monitored by thin layer chromatography on Merk’s silica plates. ^1^H and ^13^C NMR spectra were recorded on Varian 400 MHz instruments using TMS as internal standard. Mass spectrometry data were recorded on Shimadzu LCMS 2010 mass spectrometer. IR spectrometry data were recorded on a FTIR Perkin Elmer Spectrum 100 spectrometer as KBr pellets with absorption in cm^−1^.

### Single crystal x-ray diffraction of IAA

Crystals grew as clusters of colorless thin needles by slow evaporation from acetone and dioxane using a starting concentration of 5 mg/ml. Diffraction data were collected from a crystal with approximate dimensions of 0.38 × 0.06 × 0.04 mm on an Agilent Technologies SuperNova Dual Source diffractometer using a μ-focus Cu K_α_ radiation source (λ = 1.5418 Å) with collimating mirror monochromators, and at 100 K using an Oxford 700 Cryostream low temperature device. Details of crystal data, data collection, and structure refinement are listed in Tables S1–7. Data collection, unit cell refinement, and data reduction were performed using Agilent Technologies CrysAlisPro V 1.171.39.46. The structure was identified by direct methods and refined by full-matrix least-squares on F^2^ with anisotropic displacement parameters for the non-H atoms using SHELXL-2016/6^32,33^. Structure analysis was aided by using PLATON^34^ and WinGX 1.64. The hydrogen atoms on carbon were calculated in ideal positions with isotropic displacement parameters set to 1.2 × U_eq_ of the attached atoms. The hydrogen atoms bound to the nitrogen atoms were located in a ΔF map and refined with isotropic displacement parameters.

The IAA (C_9_H_6_N_2_O_4_) crystal demonstrated a monoclinic *P21/C* space group with 4 crystallographically unique molecules in the asymmetric unit. The absolute configuration was the same for all molecules in the asymmetric unit. The function, Σw(|F_o_|^2^ − |F_c_|^2^)^2^, was minimized, where w = 1/[(σ(F_o_))^2^ + (0.085 * P)^2^ + (0.0727 * P)] and P = (|F_o_|^2^ + 2|F_c_|^2^)/3. R_w_(F^2^) refined to 0.127, with R(F) equal to 0.0444 and a goodness of fit, S = 1.06. Definitions used for calculating R(F), R_w_(F^2^) and the goodness of fit, S, are given below. The data were checked for secondary extinction effects but no correction was needed. Neutral atom scattering factors and values used to calculate the linear absorption coefficient are from the International Tables for X-ray Crystallography^35^. All figures were generated using SHELXTL/PC V5.03 (Siemens Analytical X-ray Instruments, Inc., Madison, Wisconsin, USA).

### Synthetic methods

**2**,**3-dioxoindoline-4-carboxamide** (**1d**): 187 mg (0.85 mmol, 1.3 eq) of Di- Tertiary butyl carbonate and 68 mg of (0.85 mmol, 1.3 eq) of ammonium hydrogen carbonate were added to a solution of 125 mg (0.65 mmol, 1.0 eq)) of Isatin-7-acid in dioxane and maintained at 40 °C for 18 h. The solution was concentrated using a rotary evaporator. The residue was dissolved into methanol and triturated with diethyl ether to obtain 100 mg of product (80% yield) and used in subsequent reactions without further purification. ^1^H NMR (400 MHz, DMSO-*d*_6_): ^δ^ 7.52 (dd, *J* = 8.0, 1.1 Hz, 1H), 7.38 (t, *J* = 7.9 Hz, 1H), 6.92 (dd, *J* = 7.7, 1.2 Hz, 1H)^36^. 

(**E/Z**)**-3-hydrazineylidene-2-oxoindoline-4-carboxylic acid** (**1e**): 1.0 mmol of (190 mg) Isatin-7-acid was cooled to 0 °C in a 50 ml round bottom flask. Five milliliters of concentrated sulfuric acid were added to the solution, followed by 1.0 mmol (186 mg) of tosyl hydrazine. The mixture was stirred at room temperature for 3 h. Cold water was poured on the solution to allow it neutralization. After extraction with DCM (50 ml × 2), the combined organic layer was washed with water, dried over MgSO_4,_ and filtered. The filtrate was concentrated to obtain 163 mg of product in 81% yield. ^1^H NMR (400 MHz, DMSO-*d*_6_): δ δ 11.01 (s, 1H, NH), 10.79 (d, *J* = 14.6 Hz, 1H, NH), 10.00 (d, *J* = 14.5 Hz, 1H, NH), 7.38–7.31 (m, 1H), 7.29–7.20 (m, 1H), 7.02 (dt, *J* = 7.9, 1.1 Hz, 1H). ^13^C NMR (101 MHz, DMSO-*d*_6_): δ 167.81, 162.72, 139.83, 127.65, 125.73, 125.21, 123.23, 119.38, 113.33. MS (m/z): 204 (M − 1).

**1-methyl-2**,**3-dioxoindoline-4-carboxylic acid** (**1f**): 150 mg of Methyl 1- methyl-2,3-dioxoindoline-7-carboxylate (0.68 mmol) was dissolved in water: THF 1:1 (10 mL: 10 mL), to which 3.0 eq of LiOH (6.9 mmol, 60 mg) was added and stirred at ambient temperature overnight. The solution was concentrated using a rotary evaporator and the residue was dissolved in alkaline water (pH 11). Unreacted starting material was then removed by extraction using diethyl ether. The aqueous layer was then acidified with HCl and extracted twice with diethyl ether. The ether extract was washed with water and dried over MgSO_4_. A filtrate was concentrated and showed to be pure by NMR in 80% yield (113 mg). ^1^H NMR (400 MHz, DMSO-*d*_6_): ^δ^ 7.71 (t, *J* = 7.9 Hz, 1H), 7.27 (d, *J* = 7.9 Hz, 2H), 3.13 (s, 3H)^37^.

**Methyl 1-methyl-2**,**3-dioxoindoline-4-carboxylate** (**1g**): Isatin-7-acid (285 mg, 1.5 mmol) was dissolved in 20 mL of DMF, to which 2.2 eq of potassium carbonate (455 mg) was added, followed by 4.0 eq of methyl iodide. This mixture was stirred overnight at room temperature, concentrated using a rotary evaporator, and then dissolved in water. The product was extracted with dichloromethane twice, washed with water, dried over MgSO_4_, and filtered. The solution was further concentrated with a rotary evaporator to obtain an essentially pure compound in 81% yield (280 mg), ^1^H NMR (400 MHz, Chloroform-*d*): ^δ^ 7.66 (t, *J* = 7.9 Hz, 1H), 7.46 (dd, *J* = 7.9, 0.8 Hz, 1H), 7.05 (dd, *J* = 7.9, 0.9 Hz, 1H), 3.97 (s, 3H), 3.27 (s, 3H). ^13^C NMR (101 MHz, Chloroform-*d*): ^δ^ 180.16, 165.36, 157.01, 152.02, 137.71, 130.46, 124.38, 114.84, 112.83, 52.87, 26.43^38^. 

**2**,**3-dioxoindoline-7-carboxamide** (**1j**): 374 mg (1.7 mmol, 1.3 eq) of Di- Tertiary butyl carbonate and 135 mg of (1.7 mmol, 1.3 eq) of ammonium hydrogen carbonate were added to a solution of 250 mg (1.3 mmol, 1.0 eq)) of Isatin-7-acid in dioxane and maintained at 40 °C for 18 h. The solution was concentrated using a rotary evaporator and the residue was dissolved into methanol to extract the product. The solution was filtered and washed with cold methanol to obtain the 80 mg of product (33% yield) in essentially pure form and to be used in subsequent reactions without further purification. ^1^H NMR (400 MHz, DMSO-*d*6) ^δ^ 7.95 (d, *J* = 7.7 Hz, 1H), 7.51 (d, *J* = 7.1 Hz, 1H), 7.03 (t, *J* = 7.6 Hz, 1H). ^13^C NMR (101 MHz, DMSO-*d*_6_): ^δ^ 184.61, 167.63, 158.82, 151.01, 138.97, 126.26, 122.29, 121.57, 118.52. MS (m/z): 190 (M − 1).

**1-methyl-2**,**3-dioxoindoline-7-carboxylic acid** (**1k**): 500 mg of Methyl 1-methyl-2,3-dioxoindoline-7-carboxylate (2.3 mmol) were dissolved in water: THF 1:1 (25 mL: 25 mL), to which 3.0 eq of LiOH (6.9 mmol, 165 mg) was added and stirred at ambient temperature overnight. The solution was concentrated using a rotary evaporator and the residue was dissolved in alkaline water (pH 11). Unreacted starting material was then removed by extraction using diethyl ether. The aqueous layer was acidified with HCl and extracted twice with diethyl ether. The ether extract was washed with water, and then dried over MgSO_4_. The solution was filtrated and concentrated through a rotary evaporator to obtain an essentially pure compound by NMR in 83% yield (390 mg). ^1^H NMR (400 MHz, DMSO-*d*6): ^δ^ 7.81 (dd, *J* = 7.9, 1.4 Hz, 1H), 7.66 (dd, *J* = 7.3, 1.4 Hz, 1H), 7.16 (t, *J* = 7.6 Hz, 1H), 3.15 (s, 3H). ^13^C NMR (101 MHz, DMSO-*d*_6_): ^δ^ 182.60, 167.64, 159.88, 149.35, 138.13, 126.85, 123.22, 120.01, 119.01, 30.03. MS (m/z): 204 (M − 1).

**Methyl 1-methyl-2**,**3-dioxoindoline-7-carboxylate** (**1l**): Isatin-7-acid (570 mg, 3.0 mmol) was dissolved in 20 mL of DMF, to which 2.2 eq of potassium carbonate (910 mg) was added, followed by 4.0 eq of methyl iodide. This mixture was stirred overnight at room temperature. The solution was concentrated using a rotary evaporator and dissolved in water. The product was extracted with dichloromethane twice, washed with water, dried over MgSO_4_, filtered, and concentrated on rotary evaporator to obtain an essentially pure compound in 91% yield (595 mg). ^1^H NMR (400 MHz, DMSO-*d*6): ^δ^ 7.81 (dd, *J* = 7.9, 1.4 Hz, 1H), 7.69 (dd, *J* = 7.3, 1.4 Hz, 1H), 7.18 (t, *J* = 7.6 Hz, 1H), 3.88 (s, 3H), 3.09 (s, 3H). ^13^C NMR (101 MHz, DMSO-*d*6): ^δ^ 182.37, 166.41, 159.82, 149.57, 138.11, 127.31, 123.28, 120.09, 117.27, 53.25, 30.02. MS (m/z): 220 (M + 1).

(**E**,**Z**)**-3-**(**isopropylimino**)**-2-oxoindoline-7-carboxylic acid** (**1m**): Isatin-7-acid and isopropyl amine were combined in ethanol at equimolar ratio. A catalytic amount of acetic acid was added to the solution and heated to reflux for 3 h. The solution was concentrated using a rotary evaporator and then poured into water which allowed a precipitate to form. The filtrate was washed with water and dried to obtain the essentially pure E/Z mixture of product in quantitative yield. ^1^H NMR (400 MHz, DMSO-*d*_6_): ^δ^ 7.75 (dd, *J* = 7.8, 1.3 Hz, 1H), 7.69 (dd, *J* = 7.8, 1.2 Hz, 1H), 7.43 (dd, *J* = 7.4, 1.3 Hz, 1H), 6.97 (dt, *J* = 10.2, 7.6 Hz, 2H), 5.44 (hept, *J* = 6.2 Hz, 1H), 4.50 (hept, *J* = 6.3 Hz, 1H), 3.30 (hept, *J* = 6.6 Hz, 2H), 2.48 (p, *J* = 1.9 Hz, 3H), 1.27 (d, *J* = 6.2 Hz, 6H), 1.18 (dd, *J* = 6.4, 1.9 Hz, 12H). ^13^C NMR (101 MHz, DMSO-*d*_6_): ^δ^ 168.75, 168.63, 163.06, 158.85, 152.82, 151.95, 146.46, 144.52, 134.24, 133.76, 128.10, 122.83, 122.14, 121.76, 121.51, 121.22, 116.45, 52.84, 50.11, 43.13, 24.47, 23.65, 21.03.

**3-diazo-2-oxoindoline-7-carboxylic acid** (**1n**): 1 mmol of (190 mg) Isatin-7-acid in 50 ml round bottom flask was cooled to 0 °C. Sulfuric Acid was added to this 5 ml concentrate, followed by 1 mmol (186 mg) of tosyl hydrazine. A dark brown color developed immediately. The mixture was stirred at room temperature for 3 h. Upon addition to cold water, a red-colored solid precipitate developed and was recovered by filtration. The filtrate was washed with water and dried to give the product in quantitative yield (180 mg). ^1^H NMR (400 MHz, DMSO-*d*_6_) ^δ^ 10.56 (s, 1H), 7.88 (d, *J* = 7.9 Hz, 1H), 7.73 (d, *J* = 7.5 Hz, 1H), 7.12 (t, *J* = 7.8 Hz, 1H). ^13^C NMR (101 MHz, DMSO-*d*_6_): ^δ^ 166.38, 163.70, 146.34, 144.07, 135.00, 132.73, 122.74, 117.52, 113.89. FT-IR (cm^−1^): 3508, 3282, 1693, 1608, 1481, 1308, 1186. MS (m/z): 377 (M − 1).

**5-bromo-2**,**3-dioxoindoline-7-carboxylic acid** (**1o**): 382 mg of Isatin-7- Acid was dissolved in 2.0 ml of concentrated sulfuric acid at room temperature. Liquid bromine at 1.2 equivalents (195 mg) was added at room temperature. The solution was stirred while heating at 100 °C, until bromine completely dissolved in sulfuric acid (10–15 min). This was further cooled to room temperature and poured into cold water, to form an orange to yellow precipitate. This was filtered and washed with water. We obtained 520 mg of product. (96% yield.) ^1^H NMR (400 MHz, DMSO-*d*_6_): ^δ^ 10.77 (s, 1H, NH), 8.04 (d, *J* = 2.1 Hz, 1H), 7.88 (d, *J* = 2.1 Hz, 1H). ^13^C NMR (101 MHz, DMSO-*d*_6_): ^δ^ 182.26, 165.12, 159.40, 149.89, 139.48, 131.08, 121.81, 116.51, 114.12.

**Method 1-2**,**4-dioxo-1**,**4-dihydro-2H-benzo[d][1**,**3]oxazine-8-carboxamide** (**2a**): 1.0 g (5.5 mmol, 1.0 eq) of 2,3-dioxoindoline-7-carboxylic acid (1a) was added to a 50 ml round bottom flask equipped with a gas bubbler. Later 100 ml of 1 N NaOH were added dropwise. The solution was cooled to 0 °C and spiked with 10 ml of concentrated sulfuric acid. After 10 min, 410 mg (6.3 mmol, 1.2 eq) of sodium azide were added gradually over a period of 10 min. The solution was stirred at 0 °C-RT for 1 h and left at room temperature for 1 h. The reaction was then combined with 100 ml of cold water to form a precipitate. The precipitate was recovered using a Buckner flask and dried at room temperature to obtain 860 mg of product (80%). MP, 237 °C. ^1^H NMR (400 MHz, DMSO-*d*_6_) ^δ^ 12.12 (s, 1H, NH), 8.59 (s, 1H, NH), 8.28 (dd, *J* = 7.9, 1.4 Hz, 1H), 8.11 (dd, *J* = 8.1, 1.5 Hz, 2H, 1 NH), 7.33 (t, *J* = 7.8 Hz, 1H). ^1^H NMR (400 MHz, DMSO-*d*_6_ -D_2_O exchange): ^δ^ 8.21 (d, *J* = 7.8 Hz, 1H), 8.10 (dd, *J* = 7.8, 1.4 Hz, 1H), 7.32 (t, *J* = 7.9 Hz, 1H). ^13^C NMR (101 MHz, DMSO-*d*_6_): ^δ^ 169.42, 159.73, 146.39, 142.18, 136.06, 133.37, 123.04, 116.46, 112.41. FTIR (KBr pellet; ν, cm^−1^): 3408, ν(N-H), 3286 (overtone of the N-H-deformation vibration), 3180 ν(C-H)aromatic, 1778, 1726 ν(CO) anhydride, 1649 δ(N–H), 1610, 1496 ν(C C) aromatic, 1346 ν(C-O). MS (m/z): 205 (M − 1).

**Method 2-2**,**4-dioxo-1**,**4-dihydro-2H-benzo[d][1**,**3]oxazine-8-carboxamide** (**2a**): 191 mg (1.0 mmol, 1.0 eq) of 2,3-dioxoindoline-7-carboxylic acid (1a) and 240 mg of 4-Acetamido benzene sulfonyl azide were combined in a 25 ml round bottom flask. The solution was cooled to 0 °C and spiked with 2.5 ml of concentrated sulfuric acid. This was stirred at 0 °C-RT for 3 h and left at room temperature overnight. Upon mixing with 100 ml of cold water, the precipitate was recovered by filtration to yield 145 mg of product (70%).

**2-azido-3-oxo-3H-indole-7-carboxylic acid** (**2a2**): Isatin-7-acid was dissolved in DMSO and cooled to 0 °C. Sodium azide (1.2 equivalents) was added to this followed by stirring at room temperature overnight. ^1^H NMR (400 MHz, DMSO-*d*_6_): ^δ^ 10.69 (s, 1H), 8.00 (dd, *J* = 7.7, 1.4 Hz, 1H), 7.52 (dd, *J* = 7.4, 1.4 Hz, 1H), 7.04 (t, *J* = 7.6 Hz, 1H). ^13^C NMR (101 MHz, DMSO-*d*_6_): ^δ^ 184.57, 168.38, 159.19, 150.89, 139.32, 126.36, 122.31, 121.67, 118.54.

**4-aminoisoindoline-1**,**3-dione** (**2b**): Method A from Isatin-4-Acid. 191 mg (1.0 mmol) of 2,3-dioxoindoline-4-carboxylic acid, 79 mg (1.2 mmol) NaN_3_, and 2.5 mL of concentrated sulfuric acid were combined at 0 °C and and stirred for 30 min at room temperature. The product was extracted with ether, washed with water, dried over MgSO_4_, and filtered. The solution was concentrated using a rotary evaporator, yielding 151 mg of product, 90% yield. ^1^H NMR (400 MHz, DMSO-*d*_6_): ^δ^ 10.87 (s, 1H), 7.40 (dd, *J* = 8.4, 7.0 Hz, 1H), 6.93 (d, *J* = 8.4 Hz, 1H), 6.88 (d, *J* = 7.0 Hz, 1H). ^13^C NMR (101 MHz, DMSO-*d*_6_): ^δ^ 171.48, 169.87, 146.93, 135.55, 133.87, 121.49, 110.78, 110.59^18^.

**4-aminoisoindoline-1**,**3-dione  **(**2b**): Method B from Isatin-4-methyl ester. 205 mg (1.0 mmol) of 2,3-dioxoindoline-4-carboxylic acid methyl ester, 79 mg (1.2 mmol) NaN_3_, and 2.5 mL of concentrated sulfuric acid were combined and treated under similar conditions to those of method A to yield 155 mg of product, 95% yield.

**4-aminoisoindoline-1**,**3-dione  **(**2b**): Method C from Isatin-4-Amide. 43 mg (0.25 mmol) of 2,3-dioxoindoline-4-carboxamide, 20 mg (1.2 mmol) NaN_3_, and 0.8 mL of concentrated sulfuric acid were combined at 0 °C and stirred for 30 min at room temperature. The product was extracted with ether, washed with water, dried over MgSO_4_, and filtered. The solution was concentrated using a rotary, yielding 37 mg of product, 90% yield.

**4-aminoisoindoline-1**,**3-dione  **(**2b**): Method D from Isatin-4-Acid-3-Hydrazide. Fifty two mg (0.25 mmol) of 2,3-dioxoindoline-4-carboxamide, 20 mg (1.2 mmol) NaN_3_, and 0.8 mL of concentrated sulfuric acid were combined at 0 °C and stirred for 30 min at room temperature. The product was extracted with ether, washed with water, dried over MgSO_4_, and filtered. Concentration using a rotary evaporator yielded 40 mg of product, 93% yield.

**4-Methylaminoisoindoline-1**,**3-dione** (**2f**)**:** Method-A (**from 1-Methyl-2**,**3-dioxoindoline-4-carboxylic acid**): 56 mg (0.27 mmol) of 2,3-dioxoindoline-4-carboxylate, 22 mg (0.32 mmol) NaN_3_, and 1.0 mL of concentrated sulfuric acid were combined and stirred for 30 min at room temperature. The product was extracted with ether, washed with water, dried over MgSO_4_, and filtered. The solution was concentrated using a rotary evaporator, yielding 41 mg of product, 90% yield. ^1^H NMR (400 MHz, DMSO-*d*_6_): δ 10.89 (s, 1H), 7.53 (ddd, *J* = 8.5, 7.1, 0.6 Hz, 0H), 6.97–6.87 (m, 2H), 6.54 (q, *J* = 5.0 Hz, 1H, NH), 2.85 (d, *J* = 5.0 Hz, 3H). ^13^C NMR (101 MHz, DMSO-*d*_6_): δ 171.64, 169.87, 147.37, 136.32, 133.95, 116.61, 111.07, 110.03, 29.57.^19.^

**4-Methylaminoisoindoline-1**,**3-dione** (**2f**): Method-B (**from Methyl**, **1-methyl-2**,**3-dioxoindoline-4-carboxylate**)**:** 60 mg (0.27 mmol) of methyl 2,3-dioxoindoline-4-carboxylate, 22 mg (0.32 mmol) NaN_3_, and 1.0 mL of concentrated sulfuric acid were combined and stirred for 30 min at room temperature. The product was extracted with ether, washed with water, dried over MgSO_4_, and filtered. The solution was concentrated using a rotary evaporator, yielding 45 mg of product, 93% yield.

**4-amino-3-carbamoylbenzoic acid** (**2h**): 191 mg (1.0 mmol) of 2,3-dioxoindoline-5-carboxylic acid, 80 mg (1.2 mmol) of NaN_3_, and 2.5 mL of concentrated sulfuric acid were combined and stirred for 2 h at room temperature. The product was extracted with ether, washed with water, dried over MgSO_4_, and filtered. The solution was concentrated using a rotary evaporator, yielding 140 mg of product, 80% yield. ^1^H NMR (400 MHz, DMSO-*d*_6_): ^δ^ 12.37 (s, 1H, OH), 8.14 (d, *J* = 2.0 Hz, 1H), 7.96 (s, 1H, NH), 7.66 (dd, *J* = 8.7, 1.9 Hz, 1H), 7.21 (s, 2H, NH_2_), 7.14 (s, 1H, NH), 6.68 (d, *J* = 8.6 Hz, 1H). ^13^C NMR (101 MHz, DMSO-*d*_6_): ^δ^ 171.21, 167.61, 154.19, 133.33, 131.89, 116.54, 116.06, 113.31. MS (m/z): 179 (M − 1).

**Methyl 2-amino-3-carbamoylbenzoate** (**2i**): Method A. 205 mg (1.0 mmol) of methyl 2,3-dioxoindoline-7-carboxylate, 79 mg (1.2 mmol) NaN_3_, and 2.5 mL of concentrated sulfuric acid were combined and stirred for 2 h at room temperature. The product was extracted with ether, washed with water, dried over MgSO_4_, and filtered. The solution was concentrated using a rotary evaporator, yielding 183 mg of yellow solid product, 94% yield. ^1^H NMR (400 MHz, DMSO-*d*_6_): ^δ^ 8.02 (s, 2H, NH_2_), 7.93 (s, 1H, NH), 7.89 (dd, *J* = 7.9, 1.6 Hz, 1H), 7.79 (dd, *J* = 7.7, 1.6 Hz, 1H), 7.34 (s, 1H, NH), 6.55 (t, *J* = 7.8 Hz, 1H), 3.78 (s, 3H). ^13^C NMR (101 MHz, DMSO-*d*_6_): ^δ^ 171.17, 167.99, 152.04, 135.11, 135.03, 116.59, 113.66, 110.91, 52.12^39^.

**Methyl 2-amino-3-carbamoylbenzoate** (**2b**): Method B. IAA at a concentration of 0.1 mmol in methanol was stirred at reflux for 2 h, and then cooled at room temperature and concentrated. The residue was titrated with chloroform-hexane to yield a pale white solid product that was recovered by filtration in 83% yield.

**3-cyano-2-**(**methylamino**)**benzoic acid** (**2k**): 205 mg (1.0 mmol) of 1-methyl-2,3-dioxoindoline-7-carboxylic acid, 79 mg (1.2 mmol) NaN_3_, and 2.5 mL of concentrated sulfuric acid were stirred for 2 h at room temperature. The product was extracted with ether, washed with water, dried over MgSO_4_, and filtered. The solution was concentrated using a rotary evaporator, yielding 142 mg of product, 81% yield. ^1^H NMR (400 MHz, DMSO-*d*_6_): ^δ^ 8.59 (s, 1H, NH), 8.04 (dd, *J* = 7.8, 1.7 Hz, 1H), 7.71 (dd, *J* = 7.7, 1.7 Hz, 1H), 6.66 (t, *J* = 7.7 Hz, 1H), 3.21 (s, 3H). ^13^C NMR (101 MHz, DMSO-*d*_6_): ^δ^ 169.62, 153.63, 141.80, 137.45, 120.39, 115.51, 113.67, 94.21, 32.31. MS (m/z): 175 (M − 1).

**Methyl 3-cyano-2-**(**methylamino**)**benzoate** (**2l**): 219 mg (1.0 mmol) of methyl 1-methyl-2,3-dioxoindoline-7-carboxylate, 79 mg (1.2 mmol) NaN_3_, and 2.5 mL of concentrated sulfuric acid were stirred for 2 h at room temperature. The product was extracted with ether, washed with water, dried over MgSO_4_, and filtered. The solution was concentrated using a rotary evaporator, yielding 140 mg of product, 80% yield. ^1^H NMR (400 MHz, DMSO-*d*_6_): ^δ^ 8.25 (q, *J* = 5.4 Hz, 1H, NH), 8.01 (dd, *J* = 7.9, 1.7 Hz, 1H), 7.73 (dd, *J* = 7.7, 1.7 Hz, 1H), 6.67 (t, *J* = 7.8 Hz, 1H), 3.80 (s, 3H), 3.21 (d, *J* = 5.4 Hz, 3H). ^13^C NMR (101 MHz, DMSO-*d*_6_): ^δ^ 167.69, 153.07, 142.09, 136.98, 120.18, 115.64, 113.00, 94.56, 52.67, 32.44. MS (m/z): 191 (M + 1).

**5-Bromo-2**,**4-dioxo-1**,**4-dihydro-2H-benzo[d][1**,**3]oxazine-8-carboxamide** (**2o**): 536 mg (2.0 mmol, 1.0 eq) of 5-Bromo-2,3-dioxoindoline-7-carboxylic acid (1 h) was treated using procedures similar to those described under method A for producing to yield 445 mg of product (78%). ^1^H NMR (400 MHz, DMSO-*d*_6_): ^δ^ 12.00 (s, 1H, NH), 8.63 (s, 1H, NH), 8.47 (s, 1H), 8.18 (s, 2H, 1NH). ^1^H NMR (400 MHz, DMSO-*d*_6_-D_2_O exchange): δ 8.42 (d, *J* = 2.2 Hz, 1H), 8.15 (d, *J* = 2.2 Hz, 1H). ^13^C NMR (101 MHz, DMSO-*d*6): ^δ^ 168.12, 158.64, 146.03, 141.25, 138.25, 134.83, 118.52, 114.56, 114.43. MS (m/z): 261 (M-23).

**3-**((**2-carbamoylphenyl**)**amino**)**-propanoic acid** (**2p**): 219 mg (1.0 mmol) of 3-(2,3 dioxoindolin-1-yl)propanoic acid (1b), 79 mg (1.2 mmol) NaN_3_, and 2.5 mL of concentrated sulfuric acid were combined at 0 °C and and stirred for 2 h at room temperature. This was pourred into water to form a precipitate. This was collected by filtration and washed with water, and dried to obtain 200 mg of product, 95% yield. ^1^H NMR (400 MHz, DMSO-*d*_6_): ^δ^ 8.13 (t, *J* = 5.7 Hz, 1H), 7.78 (s, 1H, NH), 7.56 (dd, *J* = 7.9, 1.6 Hz, 1H), 7.25 (ddd, *J* = 8.5, 7.0, 1.5 Hz, 1H), 7.10 (s, 1H, NH), 6.66 (d, *J* = 8.4 Hz, 1H), 6.51 (t, *J* = 7.5 Hz, 1H), 3.35–3.26 (m, 4H). ^1^H NMR (400 MHz, DMSO-*d*_6_-D_2_O exchange): ^δ^ 7.53 (d, *J* = 7.8 Hz, 1H), 7.31–7.21 (m, 1H), 6.66 (d, *J* = 8.4 Hz, 1H), 6.52 (t, *J* = 7.5 Hz, 1H), 3.30 (t, *J* = 6.4 Hz, 2H), 2.48 (t, *J* = 6.3 Hz, 2H). ^13^C NMR (101 MHz, DMSO-*d*_6_): ^δ^ 173.65, 171.96, 149.57, 133.27, 129.47, 114.76, 114.37, 111.40, 38.34, 34.03. MS (m/z): 207 (M − 1), 209 (M + 1)^22^.

**2-amino-3-carbamoylbenzoic acid**, **free base** (**3a**): 1.0 g (5.5 mmol, 1.0 eq) of 2,3-dioxoindoline-7-carboxylic acid (1a) was added dropwise to a 50 ml round bottom flask equipped with gas bubbler and containing 100 ml of 1 N NaOH. The solution was cooled to 0 °C and spiked with 10 ml of concentrated sulfuric acid. After 10 min, 410 mg (6.3 mmol, 1.2 eq) of sodium azide were added gradually over a period of 10 min. The solution was stirred at 0 °C-RT for 1 h and left at room temperature for 1 h. A precipitate formed after adding the reaction to 100 ml of cold water. NaOH solution was used to adjust to pH 8. The product was recovered by filtration, washed with water and dried at room temperature to get 760 mg pale yellow solid in 80%. ^1^H NMR (400 MHz, DMSO-*d*_6_): ^δ^ 8.26 (s, 2H, NH_2_), 7.99 (s, 1H, NH), 7.83 (dd, *J* = 12.5, 7.8 Hz, 2H), 7.38 (s, 1H, NH), 6.56 (t, *J* = 7.8 Hz, 1H). ^13^C NMR (101 MHz, DMSO-*d*_6_): ^δ^ 173.27, 170.90, 152.51, 136.75, 135.26, 116.76, 113.92, 111.32. MS (m/z): 179 (M − 1).

**2-amino-5-bromo-3-carbamoylbenzoic acid** (**3b**): Method A. 268 mg of 5-Bromo-Isatin-7-acid were treated using the same conditions listed in **3a** to yield 230 mg of product (89% yield). 1H NMR (400 MHz, DMSO-*d*_6_): ^δ^ 8.07 (s, 1H, NH), 7.93 (d, *J* = 2.4 Hz, 1H), 7.90 (d, *J* = 2.5 Hz, 1H), 7.44 (s, 1H). ^13^C NMR (101 MHz, DMSO-*d*_6_): ^δ^ 169.87, 168.59, 151.21, 137.10, 136.75, 118.49, 113.64, 103.66.MS (m/z): 260 (M + 1).

**2-amino-5-bromo-3-carbamoylbenzoic acid** (**3b**): Method B. 206 mg of IAA (2b) was dissolved in 1.0 ml of concentrated sulfuric acid at room temperature. A 1.1 equivalent (180 mg) of liquid bromine was added to the solution at room temperature. This was stirred while heating at 100 °C until the bromine was completely dissolved (10–15 min). After cooling to room temperature and adding to cold water, an orange yellow precipitate formed and was recovered by filtration and washed with additional water. We obtained 240 mg of product. (93% yield).

**4 series**, Method A: A 0.1 mmol IAA in polar solvent was stirred at reflux for 2 h, cooled to room temperature, and concentrated. The residue was titrated with chloroform-hexane to generate the pale white solid product in 73–83% yield.

**4 series**, Method B: A 0.1 mmol IAA in 1.0 ml of solvent was combined with 0.2 mmol (28 mg) K_2_CO_3_ and stirred at room temperature (12 h). After complete disappearance of the IAA peak by GC, the reaction mixture was added to water and extracted with dichloromethane. This was further washed with water, dried over MgSO_4_, and recovered by filtration. 73% yield^40^.

**Ethyl 2-amino-3-carbamoylbenzoate** (**4a**): Method A. 19 mg, pale yellow solid, 78% yield. ^1^H NMR (400 MHz, DMSO-*d*_6_): ^δ^ 8.01 (s, 2H), 7.92 (s, 1H), 7.89 (dt, *J* = 8.1, 1.3 Hz, 1H), 7.78 (dt, *J* = 7.8, 1.3 Hz, 1H), 7.32 (s, 1H), 6.55 (td, *J* = 7.8, 1.0 Hz, 1H), 4.25 (qd, *J* = 7.1, 1.0 Hz, 2H), 1.29 (td, *J* = 7.1, 1.0 Hz, 3H). ^13^C NMR (101 MHz, DMSO-*d*_6_): ^δ^ 171.19, 167.58, 152.06, 135.03, 116.57, 113.65, 111.13, 60.67, 14.59. MS (m/z): 209.1 (M + 1).

**Isopropyl 2-amino-3-carbamoylbenzoate** (**4b**): Method B. 16 mg, pale yellow solid, 73% yield. ^1^H NMR (400 MHz, DMSO-*d*6): ^δ^ 8.01 (s, 2H, NH_2_), 7.92 (s, 1H, NH), 7.87 (dd, *J* = 7.9, 1.6 Hz, 1H), 7.78 (dd, *J* = 7.7, 1.6 Hz, 1H), 7.33 (s, 1H, NH), 6.54 (t, *J* = 7.8 Hz, 1H), 5.08 (hept, *J* = 6.4 Hz, 1H), 1.28 (d, *J* = 6.2 Hz, 6H). ^13^C NMR (101 MHz, DMSO-*d*_6_): ^δ^ 171.19, 167.11, 152.10, 135.01, 134.97, 116.51, 113.58, 111.43, 67.97, 22.12. MS (m/z): 223.1 (M + 1).

**S-ethyl 2-amino-3-carbamoylbenzothioate** (**5**): 0.2 mmol of ethanethiol and 0.2 mmol of K_2_CO_3_ were added to a 0.1 mmol of IAA in 1.0 ml of DMSO and stirred at room temperature. After the complete disappearance of the IAA peak by GC, the reaction mixture was poured into water. The pH was adjusted to 12 with NaOH, extracted with ether, washed with water, dried over MgSO_4_, filtered, and concentrated to obtain 20 mg, pale yellow solid, 88% yield. ^1^H NMR (400 MHz, DMSO-*d*6): ^δ^ 8.14 (s, 2H, NH_2_), 8.00 (s, 1H, NH), 7.92 (dd, *J* = 8.0, 1.4 Hz, 1H), 7.83 (dd, *J* = 7.6, 1.4 Hz, 1H), 7.38 (s, 1H, NH), 6.59 (t, *J* = 7.8 Hz, 10H), 2.95 (q, *J* = 7.4 Hz, 2H), 1.23(t, *J* = 7.3 Hz, 3H). ^13^C NMR (101 MHz, DMSO-*d*_6_): ^δ^ 192.52, 170.95, 149.78, 135.49, 133.91, 118.63, 117.01, 113.97, 23.31, 15.25. MS (m/z): 223.1 (M − 1).

**2-aminoisophthalamide** (**6**): Method A. 0.4 mmol of ammonium carbonate were added to a 0.1 mmol of IAA in 1.0 mL of dioxane and stirred at 60 °C for 4 h. The solution was concentrated in a rotary evaporator to obtain a solid product that was dissolved in 5 ml of water. The product was recovered by filtration and dried to obtain 18 mg of product in 98%. ^1^H NMR (400 MHz, DMSO-*d*_6_): ^δ^ 7.96 (s, 2H), 7.82 (s, 2H), 7.64 (d, *J* = 7.7 Hz, 2H), 7.21 (s, 2H), 6.48 (t, *J* = 7.7 Hz, 1H). ^13^C NMR (101 MHz, DMSO-*d*_6_): ^δ^ 171.47, 151.18, 132.73, 116.16, 113.14. MS (m/z): 180 (M + 1)^7^.

**2-aminoisophthalamide** (**6**): Method B. A suspension of 0.1 mmol of IAA, ammonium acetate (0.4 mmol), and acetic acid (5 mL) was heated at reflux for 2 h. The reaction mixture was cooled to room temperature and evaporated under reduced pressure. Five milliliters of water were added to the residue to form a precipitate. This was recovered by filtration, washed with water, and dried at room temperature; 16 mg of product, 95% yield.

**2-aminoisophthalamide** (**6**): Method C. A suspension of 0.1 mmol of IAA and ammonium carbonate (0.4 mmol) in DMSO was heated at 50 °C for 6 h. Water was added to form a precipitate. The precipitate was recovered by filtration, washed with water, and dried at room temperature; 16 mg of product in 95% yield.

**2-aminoisophthalamide** (**6**): Method D. A suspension of 0.1 mmol of IAA and ammonium acetate (0.4 mmol) in DMSO was heated at 50 °C for 6 h. Water was added to the residue to form a precipitate. The precipitate was recovered by filtration, washed with water, and dried at room temperature; 16 mg of product, 95% yield.

**General procedure for Compound-7 derivatives**: To a 0.1 mmol of IAA in 1.0 mL of DMSO and 0.1 mmol of amine was added and stirred at 50 °C for 6 h. The reaction was followed by GC. Upon completion, this was added to 5 ml of water and acidified to pH 2, filtered and dried. Product yield was 85–95%.

**2-amino-N-butylisophthalamide** (**7a**): 22 mg, white solid, 94% yield. ^1^H NMR (400 MHz, DMSO-*d*_6_): ^δ^ 8.29 (t, *J* = 5.7 Hz, 1H, NH), 7.82 (s, 1H, NH), 7.70 (s, 2H, NH_2_), 7.63 (dd, *J* = 7.8, 1.4 Hz, 1H), 7.54 (dd, *J* = 7.7, 1.4 Hz, 1H), 7.21 (s, 1H, NH), 6.50 (t, *J* = 7.7 Hz, 1H), 3.19 (td, *J* = 7.0, 5.5 Hz, 2H), 1.47 (dq, *J* = 7.8, 6.7, 6.3 Hz, 2H), 1.37–1.23 (m, 2H), 0.88 (t, *J* = 7.3 Hz, 3H). ^13^C NMR (101 MHz, DMSO-*d*_6_): ^δ^ 171.47, 168.92, 150.45, 132.20, 131.97, 117.86, 116.02, 113.39, 38.96, 31.60, 20.12, 14.19. MS (m/z): 236.2 (M + 1).

**2-amino-N-benzylisophthalamide** (**7b**): 25 mg, pale yellow solid, 93% yield. ^1^H NMR (400 MHz, DMSO-*d*_6_): ^δ^ 8.90 (t, *J* = 6.0 Hz, 1H, NH), 7.84 (s, 1H, NH), 7.69–7.57 (m, 2H), 7.36–7.17 (m, 6H, 1NH), 6.52 (t, *J* = 7.7 Hz, 1H), 4.41 (d, *J* = 6.0 Hz, 2H). ^13^C NMR (101 MHz, DMSO-*d*_6_): ^δ^ 171.45, 169.02, 150.62, 140.10, 132.52, 132.07, 128.72, 127.58, 127.15, 117.17, 116.17, 113.48, 42.69. MS (m/z): 270.1 (M + 1).

**2-amino-N-**(**1-propyl-1H-benzo[d]imidazol-2-yl**)**-isophthalamide**(**7c**): 32 mg, white solid, 93%^1^H NMR (400 MHz, DMSO-*d*_6_): ^δ^ 12.68 (s, 1H, NH), 8.62 (s, 2H, NH), 8.35 (dd, *J* = 7.8, 1.6 Hz, 1H), 7.83 (s, 1H, NH), 7.67 (dd, *J* = 7.7, 1.7 Hz, 1H), 7.54–7.48 (m, 2H), 7.26–7.17 (m, 3H, one NH), 6.52 (t, *J* = 7.7 Hz, 1H), 4.17 (t, *J* = 7.0 Hz, 2H), 1.81 (h, *J* = 7.3 Hz, 2H), 0.90 (t, *J* = 7.4 Hz, 3H). ^13^C NMR (101 MHz, DMSO-*d*_6_): ^δ^ 176.00, 171.87, 152.10, 151.94, 135.79, 133.04, 129.83, 129.27, 123.09, 122.94, 120.08, 116.02, 113.09, 112.43, 110.04, 43.66, 21.72, 11.63. MS (m/z): 338.2 (M + 1).

**2-amino-N-**(**5-methoxy-1-methyl-1H-benzo[d]imidazol-2-yl**)**isophthalamide** (**7d**): 33 mg, white solid, 96%. ^1^H NMR (400 MHz, DMSO-*d*_6_): ^δ^ 12.55 (s, 1H, NH), 8.61 (s, 2H, NH_2_), 8.38 (dd, *J* = 8.0, 1.6 Hz, 1H), 7.82 (s, 1H, NH), 7.67 (dd, *J* = 7.7, 1.7 Hz, 1H), 7.38 (d, *J* = 8.7 Hz, 1H), 7.18 (s, 1H, NH), 7.10 (d, *J* = 2.4 Hz, 1H), 6.86 (dd, *J* = 8.7, 2.5 Hz, 1H), 6.51 (t, *J* = 7.7 Hz, 1H), 3.76 (s, 3H), 3.63 (s, 3H). ^13^C NMR (101 MHz, DMSO-*d*_6_): ^δ^ 175.78, 171.89, 156.32, 152.17, 152.09, 135.85, 132.97, 130.06, 124.53, 120.08, 115.98, 112.98, 110.45, 110.16, 97.64, 56.04, 28.86. MS (m/z): 340.1 (M + 1).

**2-amino-N-**(**1-**(**2-morpholinoethyl**)**-1H-benzo[d]imidazol-2-yl**) **isophthalamide** (**7e**): 37 mg, white solid, 91%. ^1^H NMR (400 MHz, DMSO-*d*_6_): ^δ^ 12.65 (s, 1H, NH), 8.59 (s, 2H, NH2), 8.41–8.30 (m, 1H), 7.83 (s, 1H, NH), 7.71–7.65 (m, 1H), 7.53–7.46 (m, 2H), 7.27–7.16 (m, 3H, one NH), 6.52 (t, *J* = 7.7 Hz, 1H), 4.32 (t, *J* = 6.5 Hz, 2H), 3.45 (t, *J* = 4.7 Hz, 4H), 2.70 (t, *J* = 6.4 Hz, 2H), 2.46 (s, 4H) (merged with DMSO-d_6_ peaks). ^13^C NMR (101 MHz, DMSO-*d*_6_): ^δ^ 176.20, 171.85, 152.16, 151.98, 135.69, 133.06, 129.84, 129.29, 123.01, 122.91, 119.95, 116.02, 113.03, 112.37, 110.17, 66.59, 56.26, 53.82, 40.56. MS (m/z): 409.3 (M + 1).

**2-amino-N-**(**1-**(**tert-butyl**)**-1H-benzo[d]imidazol-2-yl**) **isophthalamide** (**7f**): 34 mg, white solid, 96%. ^1^H NMR (400 MHz, DMSO-*d*_6_): ^δ^ 12.93 (s, 1H, NH), 8.55 (s, 2H, NH_2_), 8.33 (dd, *J* = 7.8, 1.6 Hz, 1H), 7.84, (s, 1H, NH), 7.79 (dd, *J* = 7.8, 1.5 Hz, 1H), 7.68 (dd, *J* = 7.7, 1.6 Hz, 1H), 7.56 (dd, *J* = 7.8, 1.5 Hz, 1H), 7.21 (s, 1H, NH), 7.15 (pd, *J* = 7.4, 1.4 Hz, 2H), 6.54 (t, *J* = 7.7 Hz, 1H), 1.94 (s, 9H). ^13^C NMR (101 MHz, DMSO-*d*_6_): ^δ^ 176.33, 171.80, 152.68, 152.38, 135.22, 133.00, 129.63, 129.61, 122.69, 122.46, 120.17, 116.04, 114.11, 113.12, 112.43, 61.08, 30.04. MS (m/z): 352.2 (M + 1).

**2-amino-N-**(**1-**(**2-methoxyethyl**)**-1H-benzo[d]imidazol-2-yl**) **isophthalamide** (**11g**): 32 mg, pale white solid, 89%. ^1^H NMR (400 MHz, DMSO-*d*_6_): ^δ^ 12.68 (s, 1H, NH), 8.58 (s, 2H, NH_2_), 8.34 (dd, *J* = 7.8, 1.6 Hz, 1H), 7.83 (s, 1H, NH), 7.67 (dd, *J* = 7.7, 1.7 Hz, 1H), 7.55–7.45 (m, 2H), 7.29–7.14 (m, 2H, NH), 6.52 (t, *J* = 7.7 Hz, 1H), 4.37 (t, *J* = 5.3 Hz, 2H), 3.74 (t, *J* = 5.3 Hz, 2H), 3.22 (s, 3H). ^13^C NMR (101 MHz, DMSO-*d*_6_): ^δ^ 176.07, 171.87, 152.09, 151.96, 135.80, 133.06, 130.13, 129.22, 123.03, 122.92, 120.09, 115.97, 113.04, 112.31, 110.44, 69.81, 58.62, 42.16. MS (m/z): 354.1 (M + 1).

**triethylammonium 2-amino-3-cyanobenzoate** (**8a**): 0.2 mmol of triethyl amine were added to a 0.1 mmol of IAA in DMSO and heated at 50 °C for 3 h. Recorded NMR. ^1^H NMR (400 MHz, DMSO-*d*_6_): ^δ^ 8.00 (dd, *J* = 7.6, 1.7 Hz, 1H), 7.51 (s, 2H, NH2), 7.40 (dd, *J* = 7.6, 1.7 Hz, 1H), 6.50 (t, *J* = 7.6 Hz, 1H), 2.73–2.62 (m, 10H), 1.07–0.98 (m, 23H). ^13^C NMR (101 MHz, DMSO-*d*_6_): ^δ^ 170.98, 152.88, 137.19, 134.95, 120.82, 118.77, 114.78, 95.39, 45.75, 10.69.

**potassium 2-amino-3-cyanobenzoate** (**8b**): 0.1 mmol (14 mg) of K_2_CO_3_ were added To 0.1 mmol of IAA in 1.0 ml of DMSO and heated at 50 °C for 3 h. Recorded NMR. ^1^H NMR (400 MHz, DMSO-*d*_6_): ^δ^ 7.96 (dd, *J* = 7.6, 1.7 Hz, 1H), 7.30 (dd, *J* = 7.7, 1.7 Hz, 1H), 6.45 (t, *J* = 7.6 Hz, 1H). ^13^C NMR (101 MHz, DMSO-*d*_6_): ^δ^ 169.69, 153.11, 136.98, 133.84, 123.17, 119.16, 114.55, 94.77.

**Diisopropylammonium 2-amino-3-cyanobenzoate** (**8c**): 0.2 mmol of triethyl amine were added to a 0.1 mmol of IAA in DMSO and heated at 50 °C for 3 h. Recorded NMR. ^1^H NMR (400 MHz, DMSO-*d*_6_): ^δ^ 7.99 (dd, *J* = 7.6, 1.7 Hz, 1H), 7.42 (dd, *J* = 7.7, 1.7 Hz, 1H), 6.52 (t, *J* = 7.6 Hz, 1H), 3.32 (hept, *J* = 6.5 Hz, 2H), 1.22 (d, *J* = 6.4 Hz, 12H). ^13^C NMR (101 MHz, DMSO-*d*_6_): ^δ^170.19, 152.91, 137.11, 135.14, 120.51, 118.74, 114.88, 95.45, 46.26, 19.21.

**2-amino-3-cyanobenzoic acid** (**9a**): Method A. 0.2 mmol of triethyl amine were added to a 0.1 mmol of IAA in DMSO and heated at 50 °C for 3 h. The solution was poured into water and acidified to pH 2. The precipitate was recovered by filtration to obtain 16 mg of pale-yellow solid in 98% yield. ^1^H NMR (400 MHz, DMSO-*d*_6_): ^δ^ 13.24 (s, 1H, OH), 8.01 (dd, *J* = 7.9, 1.7 Hz, 1H), 7.72 (dd, *J* = 7.6, 1.7 Hz, 1H), 7.22 (s, 2H, NH_2_), 6.66 (t, *J* = 7.8 Hz, 1H). ^13^C NMR (101 MHz, DMSO-*d*_6_): ^δ^ 169.10, 152.72, 138.96, 137.29, 117.59, 115.71, 112.14, 97.12. MS (m/z): 161.2 (M − 1).

**2-amino-3-cyanobenzoic acid** (**9a**): Method B. 0.1 mmol (12 mg) of potassium butoxide were added to 0.1 mmol of IAA in 1.0 ml of DMSO and heated at 50 °C for 3 h. The reaction mixture was poured into water and acidified to pH 2. A solid product was recovered by filtration and dried to obtain a 20 mg pale-yellow pellet in 95% yield.

**2-amino-3-cyanobenzoic acid** (**9a**): Method C. 0.1 mmol (14 mg) of K_2_CO_3_ were added to 0.1 mmol of IAA in 1.0 ml of DMSO and heated at 50 °C for 3 h. The reaction was added to water and acidified to pH 2. A solid product was recovered by filtration and dried to obtain 16 mg of pale-yellow solid in 98% yield.

**2-amino-5-bromo-3-cyanobenzoic acid** (**9b**): 0.2 mmol of trimethylamine was added to a 0.1 mmol of IAA in DMSO under similar conditions to those described under 12a, method A. A 22 mg product in 93% yield. ^1^H NMR (400 MHz, DMSO-*d*_6_): ^δ^ 8.03 (d, *J* = 2.5 Hz, 1H), 7.99 (d, *J* = 2.5 Hz, 1H), 7.34 (s, 2H, NH2). ^13^C NMR (101 MHz, DMSO-*d*_6_): ^δ^ 167.95, 151.76, 140.52, 139.16, 116.26, 113.93, 104.74, 99.17. MS (m/z): 239 (M − 1).

**General procedure compound 10a–e cyano-ester Series**: Method A. 0.1 mmol (14 mg) of K_2_CO_3_ were added to 0.1 mmol of IAA in 1.0 ml of DMSO and heated at 50 °C for 3 h. After the complete disappearance of the IAA peak by GC, alkyl halide was added and stirred at room temperature for another 12 h. The reaction was extracted with dichloromethane to remove the unreacted alkyl halide and then added to water. The solution was acidified to pH 3, extracted with dichloromethane, and washed with water. The product was dried over MgSO_4_ and recovered by filtration. The filtrate was concentrated to obtain product in 80–95% yield.

**General procedure for 10a–e cyano-ester Series**: Method B.0.3 mmol of trimethylamine were added to a mixture of 0.15 mmol IAA in DMSO and heated at 50 °C for 3 h. After the complete disappearance of the IAA peak by GC, alkyl halide was added and stirred at room temperature overnight. The reaction was extracted with dichloromethane to remove the unreacted alkyl halide and then added to water. The solution was acidified to pH 3, extracted with dichloromethane, and washed with water. The product was dried over MgSO_4_ and recovered by filtration. The filtrate was concentrated to obtain product in 80–95% yield.

**General procedure compound 10a–e**, **cyano-ester**: Method C. 0.3 mmol of triethylamine were added to 0.15 mmol of IAA in 1.0 ml of DMSO and heated at 50 °C for 3 h. After the complete disappearance of the IAA peak by GC, 0.12 mmol of BOP or HBTU and 0.1 mmol of Amines/alcohols/thiols were added and stirred at room temperature overnight. The reaction was added to water and acidified. The product was filtered and washed with water, then dried over vacuum to obtain the product in 85–96% yield.

**Methyl 2-amino-3-cyanobenzoate** (**10a**): Method A. 17 mg, white solid, 95% yield. ^1^H NMR (400 MHz, DMSO-*d*_6_): ^δ^ 8.02 (dd, *J* = 8.0, 1.6 Hz, 1H), 7.76 (dd, *J* = 7.7, 1.6 Hz, 1H), 7.16 (s, 2H, NH_2_), 6.69 (t, *J* = 7.8 Hz, 1H), 3.82 (s, 3H). ^13^C NMR (101 MHz, DMSO-*d*_6_): ^δ^ 167.34, 152.31, 139.41, 136.85, 117.43, 115.91, 111.30, 97.42, 52.61. MS (m/z): 177.1 (M + 1).

**Propyl 2-amino-3-cyanobenzoate** (**10b**): Method A. 19 mg, pale white solid, 91% yield. Method B. 21 mg 93% yield. ^1^H NMR (400 MHz, DMSO-*d*_6_): ^δ^ 8.03 (dd, *J* = 7.9, 1.8 Hz, 1H), 7.76 (dd, *J* = 7.7, 1.7 Hz, 1H), 7.17 (s, 2 H, NH_2_), 6.70 (t, *J* = 7.8 Hz, 1H), 4.19 (t, *J* = 6.5 Hz, 2H), 1.69 (h, *J* = 7.0 Hz, 2H), 0.94 (t, *J* = 7.4 Hz, 3H). ^13^C NMR (101 MHz, DMSO-*d*_6_): ^δ^ 166.94, 152.38, 139.35, 136.76, 117.43, 115.92, 111.43, 97.41, 66.63, 21.93, 10.81. MS (m/z): 205.1 (M + 1).

**Prop-2-yn-1-yl 2-amino-3-cyanobenzoate** (**10c**): Method A. 25 mg, pale yellow solid, 83%. from method B, 27 mg, 90%. from method C, 28 mg, 94%. ^1^H NMR (400 MHz, DMSO-*d*_6_): ^δ^ 8.01 (dd, *J* = 8.1, 1.6 Hz, 1H), 7.79 (dd, *J* = 7.6, 1.6 Hz, 1H), 7.16 (s, 2H, NH_2_), 6.71 (t, *J* = 7.8 Hz, 1H), 4.92 (d, *J* = 2.4 Hz, 2H), 3.62 (t, *J* = 2.4 Hz, 1H). ^13^C NMR (101 MHz, DMSO-*d*_6_): ^δ^ 166.05, 152.42, 139.85, 136.88, 117.31, 116.03, 110.60, 97.59, 78.69, 78.59, 52.96. MS (m/z): 201.1 (M + 1).

**Allyl 2-amino-3-cyanobenzoate** (**10d**): Method A. 16 mg, pale white solid, 80% yield. ^1^H NMR (400 MHz, DMSO-*d*_6_): ^δ^ 8.05 (dd, *J* = 8.0, 1.6 Hz, 1H), 7.77 (dd, *J* = 7.6, 1.7 Hz, 1H), 7.17 (s, 2H, NH_2_), 6.70 (t, *J* = 7.8 Hz, 1H), 6.02 (ddd, *J* = 22.7, 10.7, 5.4 Hz, 1H), 5.37 (dq, *J* = 17.3, 1.7 Hz, 1H), 5.26 (dq, *J* = 10.5, 1.4 Hz, 1H), 4.77 (dt, *J* = 5.6, 1.5 Hz, 2H). ^13^C NMR (101 MHz, DMSO-*d*_6_): ^δ^ 166.49, 152.43, 139.50, 136.80, 132.86, 118.55, 117.39, 115.94, 111.13, 97.47, 65.55. MS (m/z): 203.1 (M + 1).

**4-methoxybenzyl 2-amino-3-cyanobenzoate** (**10e**): Method A. 26 mg, white solid, 93% yield. ^1^H NMR (400 MHz, DMSO-*d*_6_): ^δ^ 8.00 (dd, *J* = 8.0, 1.7 Hz, 1H), 7.76 (dd, *J* = 7.6, 1.6 Hz, 1H), 7.39 (d, *J* = 8.6 Hz, 2H), 7.18 (s, 2H, NH_2_), 6.94 (d, *J* = 8.6 Hz, 2H), 6.68 (t, *J* = 7.8 Hz, 1H), 5.23 (s, 2H), 3.74 (s, 3H). ^13^C NMR (101 MHz, DMSO-*d*_6_): ^δ^ 166.75, 159.72, 152.40, 139.47, 136.83, 130.56, 128.15, 117.40, 115.96, 114.35, 111.30, 97.44, 66.55, 55.57. MS (m/z): 281.1 (M − 1).

**S-ethyl 2-amino-3-cyanobenzothioate** (**11**): 0.3 mmol of triethylamine were added to 0.15 mmol of IAA in 1.0 ml of DMSO and heated at 50 °C for 3 h. After the complete disappearance of the IAA peak by GC, 0.12 mmol of HBTU and 0.1 mmol of ethanethiol were added and stirred at room temperature overnight. The reaction was added to water and acidified. The product was filtered and washed with water, then dried over vacuum to obtain 27 mg, solid, 90% yield. ^1^H NMR (400 MHz, DMSO-*d*_6_): ^δ^ 8.07 (dd, *J* = 8.1, 1.5 Hz, 1H), 7.79 (dd, *J* = 7.6, 1.5 Hz, 1H), 7.27 (s, 2H, NH_2_), 6.73 (t, *J* = 7.8 Hz, 1H), 2.99 (q, *J* = 7.3 Hz, 2H), 1.24 (t, *J* = 7.3 Hz, 3H). ^13^C NMR (101 MHz, DMSO-*d*_6_): ^δ^ 192.83, 150.04, 139.69, 135.62, 118.59, 117.18, 116.10, 97.95, 23.51, 15.06. MS (m/z): 207.1 (M + 1).

**General procedure compound 12a-g**, **cyano-amide**: 0.3 mmol of triethylamine were added to 0.15 mmol of IAA in 1.0 ml of DMSO and heated at 50 °C for 3 h. After the complete disappearance of the IAA peak by GC, 0.12 mmol of BOP or HBTU and 0.1 mmol of amines were added and stirred at room temperature overnight. The reaction was added to water and acidified. The product was filtered and washed with water, then dried over vacuum to obtain the product in 85–96% yield.

**2-amino-N-butyl-3-cyanobenzamide** (**12a**): Method C. 31 mg, white solid, 94% yield. 1H NMR (400 MHz, DMSO-*d*_6_): ^δ^ 8.48 (t, *J* = 5.6 Hz, 1H), 7.74 (dd, *J* = 7.8, 1.5 Hz, 1H), 7.58 (dd, *J* = 7.7, 1.5 Hz, 1H), 6.98 (s, 2H), 6.65 (t, *J* = 7.7 Hz, 1H), 3.20 (td, *J* = 7.0, 5.5 Hz, 2H), 1.47 (dq, *J* = 7.8, 6.5 Hz, 2H), 1.37–1.19 (m, 2H), 0.88 (t, *J* = 7.3 Hz, 3H). ^13^C NMR (101 MHz, DMSO-*d*_6_): ^δ^ 167.87, 151.48, 136.26, 133.72, 117.88, 117.12, 115.52, 96.68, 39.07, 31.46, 20.09, 14.16. MS (m/z): 218.1 (M + 1), 216.2 (M − 1).

**2-amino-N-benzyl-3-cyanobenzamide** (**12b**): Method C. 37 mg, pale yellow solid, 93% yield. ^1^H NMR (400 MHz, DMSO-*d*_6_): ^δ^ 9.25 (t, *J* = 6.0 Hz, 1H), 7.92 (dd, *J* = 7.9, 1.5 Hz, 1H), 7.60 (dd, *J* = 7.7, 1.5 Hz, 1H), 7.35–7.17 (m, 5H), 7.04 (s, 2H, NH_2_), 6.66 (t, *J* = 7.8 Hz, 1H), 4.41 (d, *J* = 5.9 Hz, 2H). ^13^C NMR (101 MHz, DMSO-*d*_6_): ^δ^ 167.99, 151.65, 139.83, 136.58, 133.98, 128.71, 127.68, 127.20, 117.85, 116.48, 115.57, 96.79, 42.76. MS (m/z): 252.1 (M + 1).

**2-Amino-3-cyano-N-**(**1-propyl-1H-benzo[d]imidazol-2-yl**) **benzamide** (**12c**): Method C. 41 mg, white solid, 91% yield. ^1^H NMR (400 MHz, DMSO-*d*_6_): ^δ^ 12.74 (s, 1H, NH), 8.42 (dd, *J* = 7.9, 1.7 Hz, 1H), 7.77 (s, 2H, NH_2_), 7.59 (dd, *J* = 7.6, 1.7 Hz, 1H), 7.54 (ddd, *J* = 7.3, 5.7, 1.7 Hz, 2H), 7.24 (pd, *J* = 7.4, 1.4 Hz, 2H), 6.67 (t, *J* = 7.7 Hz, 1H), 4.17 (t, *J* = 7.1 Hz, 2H), 1.79 (h, *J* = 7.3 Hz, 2H), 0.92 (t, *J* = 7.4 Hz, 3H). ^13^C NMR (101 MHz, DMSO-*d*_6_): ^δ^ 174.37, 152.56, 151.81, 137.24, 136.81, 129.76, 129.20, 123.33, 123.20, 120.09, 118.35, 115.38, 112.66, 110.27, 96.66, 43.82, 21.79, 11.58. MS (m/z): 320.2 (M + 1).

**2-Amino-3-cyano-N-**(**5-methoxy-1-methyl-1H-benzo[d]imidazol-2-yl**) **benzamide** (**12d**): Method C. 43 mg, white solid, 94% yield. ^1^H NMR (400 MHz, DMSO-*d*_6_): ^δ^ 12.59 (s, 1H, NH), 8.46 (dd, *J* = 7.8, 1.7 Hz, 1H), 7.70 (s, 2H, NH_2_), 7.59 (dd, *J* = 7.7, 1.7 Hz, 1H), 7.40 (d, *J* = 8.7 Hz, 1H), 7.11 (d, *J* = 2.4 Hz, 1H), 6.88 (dd, *J* = 8.7, 2.4 Hz, 1H), 6.66 (t, *J* = 7.7 Hz, 1H), 3.76 (s, 3H), 3.64 (s, 3H). ^1^H NMR (400 MHz, DMSO-*d*_6_, D_2_O exchange): ^δ^ 8.40 (dd, *J* = 7.9, 1.7 Hz, 1H), 7.55 (dd, *J* = 7.6, 1.7 Hz, 1H), 7.35 (d, *J* = 8.8 Hz, 1H), 7.08 (d, *J* = 2.4 Hz, 1H), 6.86 (dd, *J* = 8.8, 2.4 Hz, 1H), 6.66 (t, *J* = 7.7 Hz, 1H), 3.74 (s, 3H), 3.60 (s, 3H). ^13^C NMR (101 MHz, DMSO-*d*_6_): ^δ^ 174.24, 156.47, 152.53, 152.01, 137.30, 136.69, 129.99, 124.48, 120.10, 118.33, 115.33, 110.72, 110.53, 97.65, 96.61, 56.05, 28.94. MS (m/z): 322.1 (M + 1).

**2-Amino-3-cyano-N-**(**1-**(**2-morpholinoethyl**)**-1H-benzo[d]imidazol-2-yl**) **benzamide** (**12e**): Method C. 53 mg, white solid, 93% yield. ^1^H NMR (400 MHz, DMSO-*d*_6_): ^δ^ 12.71 (s, 1H, NH), 8.44 (dd, *J* = 7.9, 1.7 Hz, 1H), 7.69 (s, 2H, NH_2_), 7.60 (dd, *J* = 7.6, 1.7 Hz, 1H), 7.56–7.46 (m, 2H), 7.24 (pd, *J* = 7.5, 1.3 Hz, 2H), 6.67 (t, *J* = 7.7 Hz, 1H), 4.32 (t, *J* = 6.4 Hz, 2H), 3.44 (t, *J* = 4.5 Hz, 4H), 2.69 (t, *J* = 6.4 Hz, 2H), 2.48–2.43 (m, 3H). ^1^H NMR (400 MHz, DMSO-*d*_6_ D_2_O exchange): ^δ^ 8.41 (dd, *J* = 7.9, 1.7 Hz, 1H), 7.56 (dd, *J* = 7.5, 1.7 Hz, 1H), 7.52–7.44 (m, 2H), 7.30–7.16 (m, 2H), 6.67 (t, *J* = 7.7 Hz, 1H), 4.30 (t, *J* = 6.4 Hz, 2H), 3.41 (t, *J* = 4.7 Hz, 4H), 2.67 (t, *J* = 6.6 Hz, 2H). ^13^C NMR (101 MHz, DMSO-*d*_6_): ^δ^ 174.74, 152.61, 151.91, 137.16, 136.84, 129.80, 129.20, 123.23, 123.16, 120.00, 118.30, 115.37, 112.57, 110.42, 96.69, 66.59, 56.29, 53.81, 40.56. MS (m/z): 391.2 (M + 1).

**2-Amino-N-**(**1-**(**tert-butyl**)**-1H-benzo[d]imidazol-2-yl**)**-3-cyanobenzamide** (**12f**): Method C. 45 mg, pale white solid, 93% yield. ^1^H NMR (400 MHz, DMSO-*d*_6_): ^δ^ 8.40 (dd, *J* = 7.9, 1.7 Hz, 1H), 7.82 (dd, *J* = 7.6, 1.6 Hz, 1H), 7.59 (ddd, *J* = 10.7, 7.5, 1.8 Hz, 2H), 7.24–7.10 (m, 2H), 6.70 (t, *J* = 7.7 Hz, 1H), 1.93 (s, 9H). ^13^C NMR (101 MHz, DMSO-*d*_6_): ^δ^ 174.80, 152.83, 152.39, 136.82, 136.62, 129.65, 129.60, 122.97, 122.75, 120.06, 118.21, 115.45, 114.39, 112.69, 96.76, 61.30, 30.06. MS (m/z): 334.2 (M + 1).

**2-Amino-3-cyano-N-**(**1-**(**2-methoxyethyl**)**-1H-benzo[d]imidazol-2-yl**)**benzamide** (**12g**): Method C. 47 mg, pale white solid, 96% yield. ^1^H NMR (400 MHz, DMSO-*d*_6_): ^δ^ 8.44–8.39 (m, 1H), 7.62–7.57 (m, 1H), 7.55–7.48 (m, 2H), 7.28–7.18 (m, 2H), 6.67 (t, *J* = 7.7 Hz, 1H), 4.38 (t, *J* = 5.2 Hz, 2H), 3.72 (t, *J* = 5.2 Hz, 3H), 3.21 (s, 4H). ^13^C NMR (101 MHz, DMSO-*d*_6_): ^δ^ 174.46, 152.49, 151.73, 137.24, 136.83, 130.09, 129.14, 123.31, 123.22, 120.04, 118.30, 115.41, 112.54, 110.68, 96.65, 69.83, 58.66, 42.33. MS (m/z): 336.2 (M + 1).

**2-Amino-3**,**5-dibromobenzamide** (**13**): 206 mg of IAA (2a) were dissolved in 1.0 ml of concentrated sulfuric acid at room temperature. A 2.2 equivalent (350 mg) of liquid bromine was added to this solution at room temperature. This was stirred at 100 °C, until the bromine completely dissolved in sulfuric acid (10–15 min). The mixture was cooled to room temperature and added to cold water until a pale yellow precipitate formed. The precipitate was recovered by filtration, washed with additional water, and dried to give 280 mg of product (95% yield). ^1^H NMR (400 MHz, DMSO-*d*_6_): ^δ^ 8.03 (s, 1H, NH), 7.75 (d, *J* = 1.7 Hz, 1H), 7.70 (d, *J* = 2.0 Hz, 1H), 7.44 (s, 1H, NH), ^13^C NMR (101 MHz, DMSO-*d*6): ^δ^ 169.66, 146.39, 137.08, 131.09, 117.26, 110.80, 105.37.

**2**,**4-dioxo-1**,**2**,**3**,**4-tetrahydroquinazoline-8-carboxylic acid** (**14a**): Method A. 1.0 mmol (206 mg) of IAA (2a) was suspended in water and cooled to 0 °C. A 0.5 NaOH solution was added dropwise, until reaching pH 12. After 5 min of stirring, a clear solution was obtained. After stirring for an additional 15 min, the product was recovered by filtration, washed with water, and dried to obtain 191 mg of product in 90% yield. ^1^H NMR (400 MHz, DMSO-*d*_6_): ^δ^ 11.64 (s, 1H, NH), 10.85 (s, 1H, NH), 8.27 (dd, *J* = 7.8, 1.6 Hz, 1H), 8.17 (dd, *J* = 7.8, 1.6 Hz, 1H), 7.29 (t, *J* = 7.8 Hz, 1H). ^13^C NMR (101 MHz, DMSO-*d*_6_): ^δ^ 168.98, 162.55, 149.65, 142.02, 137.49, 133.25, 122.35, 116.17, 114.28. MS (m/z): 205 (M − 1), 207 (M + 1).

**2**,**4-dioxo-1**,**2**,**3**,**4-tetrahydroquinazoline-8-carboxylic acid** (**14a**): Method B. 0.1 mmol of IAA in DMSO was combined with 0.11 mmol (1.2 eq) of potassium tert-butoxide and stirred at room temperature overnight. The reaction mixture was poured into water and acidified to pH 2 to form a precipitate. The precipitate was recovered by filtration using a Buckner flask and dried at room temperature to obtain 20 mg of product (90%).

**2**,**4-dioxo-1**,**2**,**3**,**4-tetrahydroquinazoline-8-carboxylic acid** (**14a**): Method C 0.1 mmol of IAA in 0.6 ml of DMSO was heated at 100 °C for 1 h. The reaction mixture was poured into water and acidified to pH2 to form a precipitate. This was recovered by filtration using a Buckner flask and dried at room temperature to obtain 24 mg of product (94%).

**6-Bromo-2**,**4-dioxo-1**,**2**,**3**,**4-tetrahydroquinazoline-8-carboxylic acid** (**14b**): Method A. 0.1 mmol (285 mg) of 5-Br-IAA (2 h) were suspended in water and cooled to 0 °C, A 0.5 NaOH solution was added dropwise, until pH 12. After 5 min of stirring, a clear solution was obtained. After stirring for an additional 15 min, the product was recovered by filtration, washed with water, and dried to obtain 25 mg of product in 91% yield. 1H NMR (400 MHz, DMSO-*d*_6_): ^δ^ 8.26 (s, 2H, NH_2_), 7.99 (s, 1H, NH), 7.83 (dd, *J* = 12.5, 7.8 Hz, 2H), 7.38 (s, 1H, NH), 6.56 (t, *J* = 7.8 Hz, 1H). ^13^C NMR (101 MHz, DMSO-*d*_6_): ^δ^ 173.27, 170.90, 152.51, 136.75, 135.26, 116.76, 113.92, 111.32. MS (m/z): 283 (M − 1).

**6-Bromo-2**,**4-dioxo-1**,**2**,**3**,**4-tetrahydroquinazoline-8-carboxylic acid** (**14b**): Method B. 0.1 mmol (28.5 mg) of 5-Br-IAA (2 h) in DMSO were combined with 0.11 mmol (1.2 eq) of potassium tert-butoxide and stirred at room temperature overnight. The reaction mixture was poured into water and acidified to pH 2 to form a precipitate. This was recovered by filtration using a Buckner flask and dried at room temperature to obtain 25 mg of product (91%).

**N-**(**1-**(**tert-butyl**)**-1H-benzo[d]imidazol-2-yl**)**-2**,**4-dioxo-1**,**2**,**3**,**4-tetrahydroquinazoline-8-carboxamide** (**15**): 0.1 mmol of IAA in 1.0 ml of DMSO were heated at 100 °C for 1 h. The progress of the reaction was monitored by GC. Upon disappearance of the starting material, the solution was cooled to 0 °C. HBTU (0.15 mmol) and amine (0.16 mmol) were added and the solution was stirred at room temperature for about 12 h. The progress of the reaction was monitored by GC. The solution was poured into water (10 mL) to form a precipitate. The precipitate was recovered by filtration to obtain 55 mg of product in 94% yield. ^1^H NMR (400 MHz, DMSO-*d*_6_) δ 13.10 (s, 1H, NH), 12.43 (d, *J* = 2.2 Hz, 1H, NH), 11.51 (s, 1H, NH), 8.61 (dd, *J* = 7.8, 1.7 Hz, 1H), 8.08 (dd, *J* = 7.8, 1.7 Hz, 1H), 7.90–7.84 (m, 1H), 7.63 (dd, *J* = 7.5, 1.7 Hz, 1H), 7.31 (dd, *J* = 8.5, 7.0 Hz, 1H), 7.26–7.17 (m, 2H), 1.97 (s, 9H). ^13^C NMR (101 MHz, DMSO-*d*_6_) δ 173.58, 163.02, 152.29, 149.80, 141.96, 136.74, 131.25, 129.59, 129.48, 123.29, 123.11, 122.05, 121.40, 116.01, 114.70, 112.98, 61.67, 30.19. MS (m/z): 378.1 (M + 1).

**4-oxo-3-phenyl-2-thioxo-1**,**2**,**3**,**4-tetrahydroquinazoline-8-carboxamide** (**16**): An equimolar mixture of IAA (1.0 mmol) and amine (1.0 mmol) was added to a 10 mL vial and heated at 50 °C for about 3 h in DMSO (1 mL). The progress of the reaction was monitored by GC. Upon consumption of IAA, 1.0 mmol of PhNCS and 1.0 mmol of pyridine were added and maintained at 50 °C for about 6 h. The reaction mixture was added to water and acidified with HCl to pH 2 to form a precipitate, filtered, and dried to obtain 28 mg of product in 95% yield. ^1^H NMR (400 MHz, DMSO-*d*_6_): ^δ^ 13.65 (s, 1H, NH), 8.66 (s, 1H, NH), 8.34 (dd, *J* = 7.8, 1.4 Hz, 1H), 8.17–8.10 (m, 2H, NH), 7.47 (dd, *J* = 8.3, 6.7 Hz, 2H), 7.46–7.36 (m, 2H), 7.29 (dd, *J* = 7.2, 1.8 Hz, 2H). ^13^C NMR (101 MHz, DMSO-*d*_6_): ^δ^ 176.04, 169.50, 159.82, 139.91, 139.44, 135.06, 132.15, 129.45, 129.23, 128.77, 123.86, 118.07, 115.80. FT-IR: 1638, 1662, 1614, 1581, 1512, 1448, 1193 cm^−1^. MS (m/z): 296 (M − 1).

**3-**(**1-**(**tert-butyl**)**-1H-benzo[d]imidazol-2-yl**)**-2**,**4-dioxo-1**,**2**,**3**,**4-tetrahydroquinazoline-8-carboxamide** (**17**): An equimolar mixture of IAA (1.0 mmol) and amine (0.1 mmol) were added to a 50 mL round bottom flask and refluxed for about 12 h in acetic acid (5 mL). The progress of the reaction was monitored by GC. The contents were concentrated and added to water (10 mL) to form a precipitate. The precipitate was recovered by filtration to obtain 34 mg of product in 91% yield. ^1^H NMR (400 MHz, DMSO-*d*_6_): ^δ^ 12.71 (s, 1H), 11.37 (s, 1H), 8.24 (dd, *J* = 7.6, 1.6 Hz, 1H), 8.00 (dd, *J* = 8.0, 1.6 Hz, 1H), 7.66 (d, *J* = 8.3 Hz, 1H), 7.36 (s, 2H), 7.26 (d, *J* = 7.8 Hz, 1H), 7.18 (t, *J* = 7.7 Hz, 1H), 7.11 (t, *J* = 7.6 Hz, 1H), 7.03 (t, *J* = 7.8 Hz, 1H). ^13^C NMR (101 MHz, DMSO-*d*_6_): ^δ^ 169.61, 163.20, 152.17, 149.82, 141.99, 137.36, 131.90, 130.55, 122.74, 121.76, 121.32, 115.49, 114.51, 113.00, 59.97, 29.46. MS (m/z): 401 (M + 1 + Na).

**4-oxo-2-**(**trifluoromethyl**)**-3**,**4-dihydroquinazoline-8-carboxylic acid** (**18**): 1.0 mmol of IAA in pyridine at 0 °C was combined with 1.2 equivalent of TFAA. This was stirred at room temperature for 6 h. The reaction was added to water and acidified to pH 2 with HCl. The product was extracted with ether, washed with water, and dried over MgSO_4_. The product was recoverred after concentration to give 23 mg of product, 90% yield. ^1^H NMR (400 MHz, DMSO-*d*_6_): ^δ^ 11.84 (s, 1H), 8.20 (dd, *J* = 7.9, 1.5 Hz, 1H), 8.17 (dd, *J* = 7.8, 1.5 Hz, 1H), 7.68 (t, *J* = 7.9 Hz, 1H). ^13^C NMR (101 MHz, DMSO-*d*_6_): ^δ^ 165.77, 156.70, 156.33, 155.95, 155.58, 137.23, 137.20, 135.99, 130.47, 129.46, 120.50, 117.63, 115.96, 114.76, 112.99, 111.90. ^19^F NMR (376 MHz, dmso) δ −74.63. MS (m/z): 257 (M − 1).

**4-oxo-2-phenyl-3**,**4-dihydroquinazoline-8-carboxamide** (**19**): Method-A**:** An equimolar mixture of IAA (1.0 mmol) and benzimidine (1.0 mmol) were combined in DMSO and heated at 42 °C for 6 h. The solution was poured into water and acidified with HCl to pH 2. The product was recovered with filtration and dried to obtain 24 mg in 93% yield. ^1^H NMR (400 MHz, DMSO-*d*_6_): δ 9.73 (s, 1H, NH), 8.49 (dd, *J* = 7.6, 1.7 Hz, 1H), 8.32 (dd, *J* = 7.8, 1.7 Hz, 1H), 8.11–8.03 (m, 2H), 7.90 (S, 1H, NH), 7.60 (dddd, *J* = 13.9, 8.6, 5.7, 2.2 Hz, 4H). ^1^H NMR (400 MHz, Acetic Acid-*d*_4_): δ 8.85 (dd, *J* = 7.7, 1.7 Hz, 1H), 8.56 (dd, *J* = 7.9, 1.7 Hz, 1H), 8.23–8.17 (m, 2H), 7.73–7.60 (m, 4H). ^13^C NMR (101 MHz, DMSO-*d*_6_): δ 166.54, 162.66, 154.17, 146.70, 136.77, 133.27, 132.33, 129.97, 129.34, 129.32, 128.41, 126.42, 121.96. ^13^C NMR (101 MHz, acetic_acid-d_4_): δ 169.05, 164.56, 152.87, 147.36, 138.40, 132.41, 131.91, 131.00, 129.20, 127.75, 126.77, 126.63, 120.77. MS (m/z): 266.1 (M + 1)^31^.

**4-oxo-2-diphenyl-3**,**4-dihydroquinazoline-8-carboxamide** (**19**): Method-B: An equimolar mixture of IAA (1.5 mmol) and N-phenyl-benzamidine (1.5 mmol) was combined in DMSO and heated at 42 °C for 6 h. This was poured into water and acidified with HCl to pH 2. The product was recovered with filtration and dried to obtain 24 mg in 93% yield.

**2-**(**2-amino-3**,**6-difluorophenyl**)**-4-oxo-3**,**4-dihydroquinazoline-8-carboxamide** (**20**): IAA (1.5 mmol), 2-Amino benzimidine 2HCl (1.5 mmol), and (3.0 mmol) of potassium phosphate were combined in DMSO and heated at 80 °C for 2 h. The solution was poured into water and acidified with HCl to pH 2. The product was recovered with filtration and dried to obtain 44 mg in 95% yield. 1H NMR (400 MHz, DMSO-*d*_6_): ^δ^ 12.72 (s, 1H, NH), 9.27 (d, *J* = 3.6 Hz, 1H), 8.41 (dd, *J* = 7.6, 1.7 Hz, 1H), 8.30 (dd, *J* = 7.9, 1.7 Hz, 1H), 7.74 (d, *J* = 3.6 Hz, 1H), 7.61 (t, *J* = 7.7 Hz, 1H), 7.19 (ddd, *J* = 11.1, 8.9, 5.0 Hz, 1H), 6.43 (td, *J* = 9.3, 3.5 Hz, 1H), 6.01 (s, 2H, NH_2_). ^13^C NMR (101 MHz, DMSO-*d*_6_): ^δ^ 166.75, 162.08, 158.15, 155.75, 155.73, 148.96, 148.94, 148.53, 148.51, 146.62, 146.20, 146.18, 137.74, 137.68, 137.58, 137.52, 136.00, 130.15, 129.42, 126.77, 122.56, 117.31, 117.20, 117.10, 116.99, 108.12, 108.08, 107.92, 107.87, 101.01, 100.93, 100.76, 100.69. ^19^F NMR (376 MHz, DMSO-*d*_6_): ^δ^ −119.80, −119.81, −119.83, −119.84, −119.86, −119.87, −119.88, −137.57, −137.58, −137.60, −137.61, −137.62, −137.64, −137.65. MS (m/z): 317.1 (M + 1)

**4-oxo-2-phenyl-3**,**4-dihydroquinazoline-8-carbonitrile** (**21**): 2 mmol triethylamine were added to a mixture of 1.0 mmol IAA in DMSO and heated at 50 °C for 3 h. After cooling to room temperature, 1.2 eq of HBTU (46 mg) and 1.0 eq of benzimidine (14 mg) were added and stirred at room temperature for 6 h. The solution was poured into water and acidified to pH3. The precipitate was recovered by filtration and dried to obtain 24 mg of product in 94% yield. ^1^H NMR (400 MHz, DMSO-*d*6): ^δ^ 12.91 (s, 1H, NH), 8.38 (dd, *J* = 7.9, 1.5 Hz, 1H), 8.32 (dd, *J* = 7.5, 1.5 Hz, 1H), 8.27–8.18 (m, 2H), 7.65–7.57 (m, 4H). ^13^C NMR (101 MHz, DMSO-*d*_6_): ^δ^ 161.82, 155.08, 150.27, 139.99, 132.66, 132.32, 131.46, 129.22, 128.56, 126.84, 122.13, 117.13, 110.38. MS (m/z): 248 (M + 1)^31^.

### One sentence summary

Discovery of isatoic anhydride-8-amide enables easy synthesis of substituted quinazolines and highly substituted anilines.

## Supplementary information

Supplementary materials

## Data Availability

Crystallographic model data is available through the CCDC under identifier 1896630. Requests for materials should be addressed to K.D.W. (kenneth.westover@utsouthwestern.edu).

## References

[1] Connolly DJ, Cusack D, O’Sullivan TP, Guiry PJ (2005). Synthesis of quinazolinones and quinazolines. Tetrahedron.

[2] Marzaro G, Guiotto A, Chilin A (2012). Quinazoline derivatives as potential anticancer agents: a patent review (2007–2010). Expert Opin Ther Pat.

[3] Sequist LV (2013). Phase III study of afatinib or cisplatin plus pemetrexed in patients with metastatic lung adenocarcinoma with EGFR mutations. J Clin Oncol.

[4] Liu KK (2011). Quinazolines with intra-molecular hydrogen bonding scaffold (iMHBS) as PI3K/mTOR dual inhibitors. Bioorg Med Chem Lett.

[5] Asif M (2014). Chemical characteristics, synthetic methods, and biological potential of quinazoline and quinazolinone derivatives. Int J Med Chem.

[6] Ajani OO, Aderohunmu DV, Umeokoro EN, Olomieja AO (2016). Quinazoline pharmacophore in therapeutic medicine. Bangl J Pharmacol.

[7] Clark AS (1995). Antitumor imidazotetrazines. 32. Synthesis of novel imidazotetrazinones and related bicyclic heterocycles to probe the mode of action of the antitumor drug temozolomide. J Med Chem.

[8] Perry, R. J., Marrese, C. A. & Tandon, S. Use of 2,1-benzisoxazol-3 (1H)-ones as antioxidants in color photographic processing methods. 5,554,493 (1996).

[9] Lo Conte M, Carroll KS (2012). Chemoselective ligation of sulfinic acids with aryl-nitroso compounds. Angew Chem Int Ed Engl.

[10] Chandregowda V, Rao GV, Reddy GC (2007). Convergent approach for commercial synthesis of gefitinib and erlotinib. Org Process Res Dev.

[11] Anderson, M. B. *et al*. Preparation of 4-arylamino-quinazolines as activators of caspases and inducers of apoptosis. US20100069383A1 (2010).

[12] Tiwary B, Pradhan K, Nanda A, Chakraborty R (2015). Implication of quinazoline-4 (3H)-ones in medicinal chemistry: a brief review. J Chem Biol Therapeutics.

[13] Wakelin LP (2003). Bisintercalating threading diacridines: relationships between DNA binding, cytotoxicity, and cell cycle arrest. J Med Chem.

[14] Edwards, W. G. H. & Petrow, V. Some Examples of the Schmidt Rearrangement. *J Chem Soc* **3**, 1713–1714 (1948). 18101490

[15] Edwards WGH, Petrow V (1948). Some Examples of the Schmidt Rearrangement. J Chem Soc.

[16] Wang J (2015). Design, synthesis and biological evaluation of pyridazino[3,4,5-de]quinazolin-3(2H)-one as a new class of PARP-1 inhibitors. Bioorg Med Chem Lett.

[17] Chattopadhyay G, Ray PS (2011). Hydrazine-hydroquinone complex as an efficient solid phase hydrazine donor: high yield synthesis of luminol and isoluminol. J Chem Res.

[18] Muthuppalniappan, M., Kumar, S., Thomas, A., Khairatkar-Joshi, N. & Mukhopadhyay, I. Preparation of phthalimide derivatives as TRPA1 modulators. WO2009118596A2 (2009).

[19] Dietrich, G.K & Manfred, D. Constitution and chemiluminescence. I. Sterical resonance hindrance in alkylated aminophthalylhydrazides.*Chemische Berichte* **95**, 2018-2026 (1962).

[20] Fidalgo, J. D. V. *et al*. Preparation of quinolone derivatives as antibacterials that inhibit bacterial DNA gyrase. WO2016020836A1 (2016).

[21] Deligeorgiev T, Vasilev A, Vaquero JJ, Alvarez-Builla J (2007). A green synthesis of isatoic anhydrides from isatins with urea–hydrogen peroxide complex and ultrasound. Ultrasonics sonochemistry.

[22] Ozaki K, Yamada Y, Oine T (1983). Studies on 4 (1H)-quinazolinones. III. Some derivatizations of 2-ethoxycarbonylalkyl-1-substituted-4 (1H)-quinazolinones. Chem Pharm Bull.

[23] Cheng R, Guo TJ, Zhang-Negrerie D, Du YF, Zhao K (2013). One-Pot Synthesis of Quinazolinones from Anthranilamides and Aldehydes via p-Toluenesulfonic Acid Catalyzed Cyclocondensation and Phenyliodine Diacetate Mediated Oxidative Dehydrogenation. Synthesis-Stuttgart.

[24] Malinowski Z (2015). Synthesis and biological evaluation of some amino- and sulfanyl-3H-quinazolin-4-one derivatives as potential anticancer agents. Monatsh Chem.

[25] Bao Y (2015). Copper-catalyzed radical methylation/C-H amination/oxidation cascade for the synthesis of quinazolinones. J Org Chem.

[26] Jia F-C, Zhou Z-W, Xu C, Wu Y-D, Wu A-X (2016). Divergent Synthesis of Quinazolin-4 (3H)-ones and Tryptanthrins Enabled by a tert-Butyl Hydroperoxide/K_3_PO_4_-Promoted Oxidative Cyclization of Isatins at Room Temperature. Org Lett.

[27] Gray J, Waring D (1980). 3‐Amino‐2, 1‐benzoisothiazole. Synthesis of some chloro and trifluoromethyl derivatives. Journal of Heterocyclic Chemistry.

[28] Staiger R, Wagner E (1948). Isatoic anhydride. II. Reactions of isatoic anhydride with ammonia. J Org Chem.

[29] Wang G, Chen X, Deng Y, Li Z, Xu X (2015). Synthesis and Nematicidal Activities of 1,2,3-Benzotriazin-4-one Derivatives against Meloidogyne incognita. J Agric Food Chem.

[30] Zhang H (2018). Design, synthesis and biological activities of 2,3-dihydroquinazolin-4(1H)-one derivatives as TRPM2 inhibitors. Eur J Med Chem.

[31] Buhrmann M, Hardick J, Weisner J, Quambusch L, Rauh D (2017). Covalent Lipid Pocket Ligands Targeting p38alpha MAPK Mutants. Angew Chem Int Ed Engl.

[32] Sheldrick GM (2015). SHELXT-Integrated space-group and crystal-structure determination. Acta Crystallographica Section A: Foundations and Advances.

[33] Sheldrick GM (2015). Crystal structure refinement with SHELXL. Acta Crystallographica Section C: Structural Chemistry.

[34] Spek A (2003). Single-crystal structure validation with the program PLATON. Journal of Applied Crystallography.

[35] Prince, E. & Wilson, A. J. C. International tables for crystallography (2004).

[36] Bramson HN (2001). Oxindole-based inhibitors of cyclin-dependent kinase 2 (CDK2): design, synthesis, enzymatic activities, and X-ray crystallographic analysis. J Med Chem.

[37] Zeng R, Dong G (2015). Rh-Catalyzed decarbonylative coupling with alkynes via C-C activation of isatins. J Am Chem Soc.

[38] Bredenkamp A, Mohr F, Kirsch SF (2015). Synthesis of Isatins through Direct Oxidation of Indoles with IBX-SO3K/NaI. Synthesis-Stuttgart.

[39] Holmes JL (2016). Synthesis of Novel Hydroxymethyl-Substituted Fused Heterocycles. Synthesis-Stuttgart.

[40] Gustavsson, A., Jendeberg, L., Roussel, P., Slater, M. & Thor, M. New Compounds. WO 03/004458A1 (2003).

