# Publisher Correction: Green Synthesis of Substituted Anilines and Quinazolines from Isatoic Anhydride-8-amide

**DOI:** 10.1038/s41598-019-55969-z

**Published:** 2019-12-30

**Authors:** Sudershan R. Gondi, Asim K. Bera, Kenneth D. Westover

**Affiliations:** Departments of Biochemistry and Radiation Oncology, The University of Texas Southwestern Medical Center at Dallas, Dallas, Texas 75390 United States

Correction to: *Scientific Reports* 10.1038/s41598-019-50776-y, published online 03 October 2019

The original version of this Article contained an error in Table 1, where parts of the table had been incorrectly duplicated and appeared as a separate table.

This error has now been corrected in the PDF and HTML versions of the Article.

