# Supplementary material for: Green Synthesis of Substituted Anilines and Quinazolines from Isatoic Anhydride-8-amide
Source: Sci Rep. 2019 Oct 3;9:14258. doi: 10.1038/s41598-019-50776-y (PMC6776664; doi:10.1038/s41598-019-50776-y)
Supplement: Supplementary file 1 — Supplementary materials [file 41598_2019_50776_MOESM1_ESM.docx]

Supplementary Materials for

Green Synthesis of Substituted Anilines and Quinazolines from Isatoic Anhydride-8-amide

Sudershan Gondi, Asim Bera, Kenneth D. Westover.

Correspondence to: kenneth.westover@utsouthwestern.edu

**Fig. S1.** Examples of FDA approved and tool compounds incorporating the quinazoline pharmacophore.


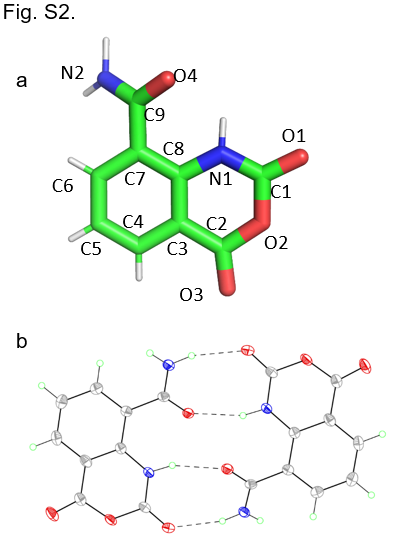


Fig. S2. IAA crystal structure. (A) Numbering scheme for coordinates. (B) H-bonding in IAA crystals. Displacement ellipsoids are scaled to the 50% probability level.


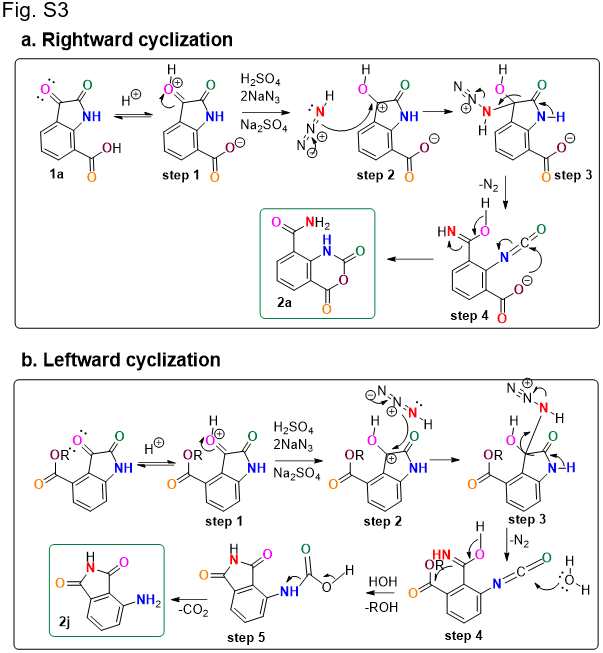


Fig. S3. Mechanisms of (A) rightward (clockwise) and (B) leftward (counterclockwise) cyclization reactions related to formation of IAA.

Fig. S4. Proposed mechanism of cyano derivative substituted anilines.


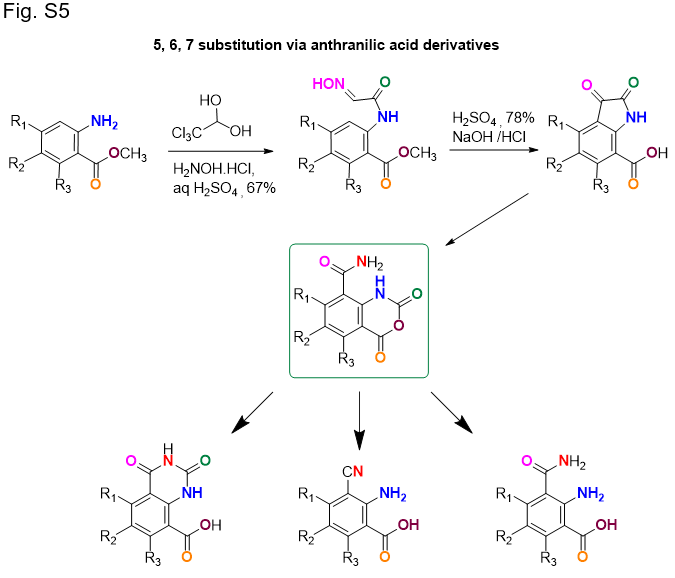


**Fig. S5**. Strategy for obtaining substituted quinazolines and substituted anilines from anthranilic acid derivatives.

Table S1. Crystal data and structure refinement for IAA

| Table S1. Crystal data and structure refinement for IAA | |  |
| --- | --- | --- |
| Empirical formula | C_9_ H_6_ N_2_ O_4_ |  |
| Formula weight | 206.16 |  |
| Temperature | 100 K |  |
| Wavelength | 1.54184 Å |  |
| Crystal system | monoclinic |  |
| Space group | P 21/c |  |
| Unit cell dimensions | a = 7.6432 Å | α = 90 ° |
|  | b = 4.8714 Å | β = 95.006 ° |
|  | c = 22.3617 Å | γ = 90 ° |
| Volume | 829.42(6) Å^3^ |  |
| Z | 4 |  |
| Density (calculated) | 1.651 Mg/m^3^ |  |
| Absorption coefficient | 1.145 mm^-1^ |  |
| F(000) | 424 |  |
| Crystal size | 0.380 x 0.060 x 0.040 mm^3^ |  |
| Theta range for data collection | 3.969 to 68.769°. |  |
| Index ranges | -9<=h<=8, -5<=k<=5, -26<=l<=26 |  |
| Reflections collected | 6181 |  |
| Independent reflections | 1521 [R(int) = 0.0439] |  |
| Completeness to theta = 67.684° | 100.00% |  |
| Absorption correction | Semi-empirical from equivalents |  |
| Max. and min. transmission | 1.00 and 0.743 |  |
| Refinement method | Full-matrix least-squares on F^2^ |  |
| Data / restraints / parameters | 1521 / 0 / 148 |  |
| Goodness-of-fit on F^2^ | 1.056 |  |
| Final R indices [I>2sigma(I)] | R1 = 0.0444, wR2 = 0.1206 |  |
| R indices (all data) | R1 = 0.0513, wR2 = 0.1267 |  |
| Extinction coefficient | n/a |  |
| Largest diff. peak and hole | 0.311 and -0.293 e.Å^-3^ |  |
|  |  |  |
| Rw(F^2^) = {Sw(\|Fo\|^2^ -\|Fc\|^2^)^2^/Sw(\|Fo\|)^4^}^1/2^ where w is the weight given each reflection. R(F) = S(\|Fo\| - \|Fc\|)/S\|Fo\|} for reflections with Fo > 4(s(Fo)). S = [Sw(\|Fo\|^2^ - \|Fc\|^2^)^2^/(n - p)]^1/2^, where n is the number of reflections and p is the number of refined parameters | |  |

Data S1. CCDC 1896630 file. Coordinates file for IAA x-ray structure.

| Table S2. Atomic coordinates (x 10^4^) and equivalent isotropic displacement parameters (Å^2^ x 10^3^) for IAA. Ueq is defined as one third of the trace of the orthogonalized Uij tensor. | | | | | | |
| --- | --- | --- | --- | --- | --- | --- |
| ________________________________________________________________________________ | | | | | | |
|  | x | y | z | Ueq |  |  |
| ________________________________________________________________________________ | | | | | | |
| C1 | 8930(2) | 4323(3) | 4332(1) | 18(1) |  |  |
| C2 | 9828(2) | 7162(4) | 3511(1) | 21(1) |  |  |
| C3 | 8120(2) | 8537(3) | 3491(1) | 20(1) |  |  |
| C4 | 7705(2) | 10581(4) | 3064(1) | 22(1) |  |  |
| C5 | 6104(2) | 11891(4) | 3048(1) | 24(1) |  |  |
| C6 | 4916(2) | 11170(3) | 3460(1) | 20(1) |  |  |
| C7 | 5295(2) | 9143(3) | 3892(1) | 16(1) |  |  |
| C8 | 6929(2) | 7783(3) | 3902(1) | 17(1) |  |  |
| C9 | 3977(2) | 8333(3) | 4317(1) | 17(1) |  |  |
| N1 | 7408(2) | 5741(3) | 4318(1) | 17(1) |  |  |
| N2 | 2890(2) | 10246(3) | 4480(1) | 20(1) |  |  |
| O1 | 9325(2) | 2429(2) | 4664(1) | 21(1) |  |  |
| O2 | 10107(2) | 5093(3) | 3934(1) | 21(1) |  |  |
| O3 | 10988(2) | 7621(3) | 3197(1) | 28(1) |  |  |
| O4 | 3941(2) | 5927(2) | 4504(1) | 19(1) |  |  |
| ________________________________________________________________________________ | | | | | | |
|  |  |  |  |  |  |  |
